# Genetic Association Analysis of Common Variants in *FOXO3* Related to Longevity in a Chinese Population

**DOI:** 10.1371/journal.pone.0167918

**Published:** 2016-12-09

**Authors:** Rong Lin, Yunxia Zhang, Dongjing Yan, Xiaoping Liao, Xianshou Wang, Yunxin Fu, Wangwei Cai

**Affiliations:** 1 Department of Biology, Hainan Medical College, Haikou, Hainan, China; 2 Department of Biochemistry and Molecular Biology, Hainan Medical College, Haikou, Hainan, China; 3 Department of Neurology, the First Affiliated Hospital of Hainan Medical College, Haikou, Hainan, China; 4 Specialized Biotechnologies Lab, Hainan Medical College, Haikou, Hainan, China; 5 Division of Biostatistics and Human Genetics Center, The University of Texas Health Science Center at Houston, Houston, Texas, United States of America; 6 Laboratory for Conservation and Utilization of Bio-Resources, Yunnan University, Kunming, Yunnan, China; Kunming Institute of Zoology, Chinese Academy of Sciences, CHINA

## Abstract

Recent studies suggested that forkhead box class O3 (FOXO3) functions as a key regulator for the insulin/insulin-like growth factor-1signaling pathway that influence aging and longevity. This study aimed to comprehensively elucidate the association of common genetic variants in *FOXO3* with human longevity in a Chinese population. Eighteen single-nucleotide polymorphisms (SNPs) in *FOXO3* were successfully genotyped in 616 unrelated long-lived individuals and 846 younger controls. No nominally significant effects were found. However, when stratifying by gender, four SNPs (rs10499051, rs7762395, rs4946933 and rs3800230) previously reported to be associated with longevity and one novel SNP (rs4945815) showed significant association with male longevity (*P*-values: 0.007–0.032), but all SNPs were not associated with female longevity. Correspondingly, males carrying the ***G***-G-***T***-***G*** haplotype of rs10499051, rs7762395, rs4945815 and rs3800230 tended to have longer lifespan than those carrying the most common haplotype A-G-C-T (odds ratio = 2.36, 95% confidence interval = 1.20–4.63, *P* = 0.013). However, none of the associated SNPs and haplotype remained significant after Bonferroni correction. In conclusion, our findings revealed that the *FOXO3* variants we tested in our population of Chinese men and women were associated with longevity in men only. None of these associations passed Bonferroni correction. Bonferroni correction is very stringent for association studies. We therefore believe the effects of these nominally significant variants on human longevity will be confirmed by future studies.

## Introduction

Human longevity is a complex and multifactorial phenotype influenced by various environmental and genetic factors [1]. The genetic component of human lifespan variation has been estimated to be approximately 25% in twin studies [2, 3]. Which genes can extend lifespan? So far, many studies of candidate longevity-associated genes were performed to address this question in humans.

Forkhead box class O3 (FOXO3) is an evolutionarily conserved transcription factor and a key regulator in the insulin/insulin-like growth factor-1 (IGF-1) signaling (IIS) pathway [4]. It regulates expression of genes controlling a multitude of processes, such as stress resistance, metabolism, cell cycle arrest, and apoptosis, that could enhance health and lifespan [4–6]. Effective control of FOXO3 in response to environmental stimuli probably plays a vital role in preventing aging and age-related diseases including cardiovascular disease, type 2 diabetes, cancer and neurodegenerative diseases [6]. *FOXO3* genotype is an important risk factor for mortality, particularly coronary artery disease mortality in older populations [7, 8]. *FOXO3* is one of the homologues of dauer formation-16 (*daf-16*) in *C*. *elegans*. DAF-16 and its orthologues in other model organisms such as *Drosophila* and mice have been demonstrated to influence metabolism and lifespan [4, 6]. These aspects render *FOXO3* a quite likely candidate gene for human longevity.

In 2008, Willcox *et al*. [9] investigated three *FOXO3* genetic variations and first reported a strong association between the three variations and longevity in American men of Japanese and Okinawan descent. Half a year later, this association was validated in men but not in women by a southern Italian centenarian study [10]. These findings suggest that *FOXO3* may be involved in determining human longevity, especially in men. A number of case-control studies have explored the association between *FOXO3* polymorphisms and longevity in populations of diverse ancestry, such as Japanese [9], Italian [10], German [11], French [11] and Chinese [12] populations. However, some of these studies reported inconsistent results.

In this study, we performed a case-control study to explore the possible association of common variants in the *FOXO3* gene with human longevity, and to clarify any gender-specific effects of these polymorphisms, in a Chinese population. Further, in addition to four previously reported longevity-associated single-nucleotide polymorphisms (SNPs) and one SNP in 5' flanking region, thirteen tagging SNPs were included in order to cover more of the known common variants of *FOXO3*.

## Materials and Methods

### Study population

A total of 1,462 unrelated Chinese participants (616 long-lived individuals (LLIs) and 846 younger controls) were enrolled from Hainan Island between 2009–2014. All LLIs were greater than or equal to 98 years of age at the time of recruitment (mean age: 102.4±2.3 years). The LLIs comprised 578 (93.8%) Han and 38 (6.2%) Li people and 83.4% of the LLIs were females. The control participants were between 30 and 70 years of age (mean age: 48.9±10.6 years) and matched the LLIs by geographical origin. The controls comprised 777 (91.8%) Han and 69 (8.2%) Li people and 81.2% of the controls were females. The study was approved by the Ethics Committee of Hainan Medical College and the local data protection authorities, and was conducted according to The Code of Ethics of the World Medical Association (Declaration of Helsinki). All participants provided informed, written consent prior to participation.

### SNP selection and genotyping

Thirteen tagging SNPs, which span *FOXO3* (chromosome 6: 108,554,835–108,689,774 134.94 kbp, human genome reference assembly GRCh38/hg38), were selected from the phase III Han Chinese in Beijing (CHB) and Southern Han Chinese (CHS) populations based on *r*^2^ greater than 0.90 and minor allele frequency (MAF) ≥0.05 (Table 1). Another four SNPs (rs10499051, rs2802292, rs9400239 and rs479744), which were previously reported to be associated with human longevity [10, 11, 13], and one SNP in 5' flanking region (rs7746906) were selected. All SNPs were genotyped with a custom-by-design 48-Plex SNPscan^™^ Kit (Cat#:G0104; Genesky Biotechnologies Inc., Shanghai, China). As presented by Chen *et al*. [14], it was based on double ligation and multiplex fluorescence polymerase chain reactions and developed according to patented SNP genotyping technology by Genesky Biotechnologies Inc. In order to reduce artifacts due to batch or plate position effects, case and control samples were interspersed randomly within 96-well plates and genotypes were read blind to the case or control status. Furthermore, 78 blind duplicate samples were distributed across all genotyping plates to validate genotyping and each plate contained one negative control.

**Table 1:** Primary information for genotyped SNPs.

No.	dbSNP ID	Chromosome position^a^	Location in gene region	Call rate	HWE (controls)
1	rs768024	Chr 6:108554905	5'-flanking	100%	0.095
2	rs9486902	Chr 6:108556849	5'-flanking	99.1%	0.35
3	rs7746906	Chr 6:108557610	5'-flanking	99.7%	0.095
4	rs10499051^b^	Chr 6:108580977	intron	100%	0.38
5	rs12206094	Chr 6:108584997	intron	99.9%	0.93
6	rs2802292^b^	Chr 6:108587315	intron	99.0%	0.65
7	rs13220810	Chr 6:108591998	intron	99.9%	0.64
8	rs2764261	Chr 6:108606639	intron	100%	0.54
9	rs3813498	Chr 6:108622962	intron	99.7%	0.81
10	rs7762395	Chr 6:108623904	intron	100%	0.19
11	rs13207511	Chr 6:108632772	intron	100%	0.66
12	rs9400239^b^	Chr 6:108656460	intron	100%	0.6
13	rs4946933	Chr 6:108659914	intron	99.9%	0.26
14	rs4945815	Chr 6:108670603	intron	99.9%	0.27
15	rs3800229	Chr 6:108675760	intron	99.5%	0.65
16	rs3800230	Chr 6:108676925	intron	100%	0.42
17	rs1159806	Chr 6:108685635	3'-flanking	100%	0.94
18	rs479744^b^	Chr 6:108698829	3'-flanking	99.9%	0.8

HWE indicates Hardy-Weinberg equilibrium.

^a^ Chromosome position determined by human genome reference assembly GRCh38/hg38.

^b^ SNPs selected from previous reports.

The call rate for each SNP was above 98% (Table 1). The concordance for duplicate samples was >99%, and all 18 *FOXO3* SNPs were in Hardy–Weinberg equilibrium (HWE) in the control group (all *P*>0.05).

### Statistical analysis

All analyses were performed using the web-based tool SNPStats (http://bioinfo.iconcologia.net/snpstats/start.htm) [15]. Departure from HWE was checked for each SNP in the control group using exact test. Odds ratios (ORs) and 95% confidence intervals (CIs) for association of individual SNPs were assessed using logistic regression models. Haplotype frequencies were inferred using an implementation of the expectation-maximization algorithm. Linkage disequilibrium (LD) between SNPs was analyzed using the pairwise LD measure D' and *r*^2^. The statistic D'<0.20 indicates no LD, D'>0.50 LD, D'>0.80 strong LD and D' = 1 complete LD. The statistic *r*^2^<0.50 indicates low LD, *r*^2^>0.50 moderate high LD, *r*^2^>0.80 high LD and *r*^2^ = 1 perfect LD. The association parameters of human longevity were estimated for each haplotype by comparison to the most frequent haplotype. Effects of rare haplotypes (frequency <1%) on human longevity were evaluated after lumping them together. Another web-based tool SHEsis [16] (http://analysis.bio-x.cn/myAnalysis.php) was also used to produce similar results compared with SNPStats. All *P*-values presented are two sided, and *P*<0.05 were considered significant in the initial analyses. *P* values obtained were corrected (*P*c) for multiple testing using Bonferroni for the number of tests.

## Results

### Single-SNP association analysis of *FOXO3* variants with human longevity

Table A in S1 File represents the single-SNP associations between the 18 SNPs and longevity. The genotype and allele frequencies for the 18 SNPs did not show any significant difference between the LLIs and younger controls (all *P*>0.05). We subsequently performed a sex-stratified analysis and observed that 5 *FOXO3* intronic SNPs were nominal significant associated with human longevity in men only **(**Table 2 and Table B in S1 File). In particular, the minor alleles of SNPs rs10499051 (OR = 1.96, 95% CI = 1.06–3.62, *P* = 0.032), rs4946933 (OR = 1.96, 95% CI = 1.06–3.62, *P* = 0.032), rs4945815 (OR = 2.43, 95% CI = 1.26–4.68, *P* = 0.007) and rs3800230 (OR = 1.55, 95% CI = 1.05–2.28, *P* = 0.025) increased the probability of male longevity while the minor allele of SNP rs7762395 decreased the probability of male longevity (OR = 0.32, 95% CI = 0.11–0.96, *P* = 0.024). However, all these results did not hold up for correction for multiple testing.

**Table 2:** Genotype and allele frequencies of longevity-associated *FOXO3* polymorphisms in the long-lived individuals and controls.

SNP NO.	dbSNP ID	Genotype/Allele	LLIs	Controls	OR(95%CI)	*P*	*Pc*
Men							
SNP 4	rs10499051	A/A	77 (75.5%)	136 (85.5%)	1.00	0.067	1
		G/A	24 (23.5%)	23 (14.5%)	1.84 (0.98–3.48)		
		G/G	1 (1%)	0 (0%)			
		A	178 (87.3%)	295 (92.8%)	1.00		
		G	26 (12.7%)	23 (7.2%)	1.96 (1.06–3.62)	0.032	0.576
SNP 10	rs7762395	G/G	98 (96.1%)	141 (88.7%)	1.00	0.071	1
		G/A	4 (3.9%)	17 (10.7%)	0.34 (0.11–1.04)		
		A/A	0 (0%)	1 (0.6%)			
		G	200 (98.0%)	299 (94.0%)	1.00		
		A	4 (2.0%)	19 (6.0%)	0.32 (0.11–0.96)	0.024	0.432
SNP 13	rs4946933	G/G	77 (75.5%)	136 (85.5%)	1.00	0.067	1
		G/A	24 (23.5%)	23 (14.5%)	1.84 (0.98–3.48)		
		A/A	1 (1%)	0 (0%)			
		G	178 (87.3%)	295 (92.8%)	1.00		
		A	26 (12.7%)	23 (7.2%)	1.96 (1.06–3.62)	0.032	0.576
SNP 14	rs4945815	C/C	78 (76.5%)	141 (88.7%)	1.00	0.02	0.36
		C/T	23 (22.5%)	18 (11.3%)	2.31 (1.17–4.54)		
		T/T	1 (1%)	0 (0%)			
		C	179 (87.7%)	300 (94.3%)	1.00		
		T	25 (12.3%)	18 (5.7%)	2.43 (1.26–4.68)	0.007	0.126
SNP 16	rs3800230	T/T	43 (42.2%)	86 (54.1%)	1.00	0.077	1
		G/T	46 (45.1%)	63 (39.6%)	1.46 (0.86–2.48)		
		G/G	13 (12.8%)	10 (6.3%)	2.60 (1.05–6.41)		
		T	132 (64.7%)	235 (73.9%)	1.00		
		G	72 (35.3%)	83 (26.1%)	1.55 (1.05–2.28)	0.025	0.45

Values shown are only for polymorphisms with *P*<0.05 in logistic regression analyses using SNPStats. *P*c, *P* values after Bonferroni correction using the number of SNPs tested (n = 18).

Of note, the male LLIs had significantly higher MAFs of SNPs rs10499051 (12.7% vs 6.5%, *P* = 0.0037), rs4946933 (12.7% vs 6.4%, *P* = 0.0032), rs4945815 (12.3% vs 5.2%, *P* = 0.0004) and rs3800230 (35.3% vs 25.3%, *P* = 0.0038) and lower MAF of rs7762395 (2.0% vs 6.7%, *P* = 0.0041) as compared to the female LLIs whereas the male and female controls had similar MAFs of these five SNPs (*P* = 0.37–0.77) (Table C in S1 File). In other words, the minor alleles of rs10499051, rs4946933, rs4945815 and rs3800230 were male-specific enriched while the minor allele of rs7762395 was male-specific depleted as the populations age. Bonferroni correction is very stringent for genetic association analysis. It is therefore nevertheless likely that the minor alleles of SNPs rs10499051, rs4946933, rs4945815 and rs3800230 may be beneficial to male longevity while the minor allele of rs7762395 may be harmful to male longevity.

### Linkage disequilibrium and haplotype association analysis of *FOXO3* variants with human longevity

**Fig 1:**
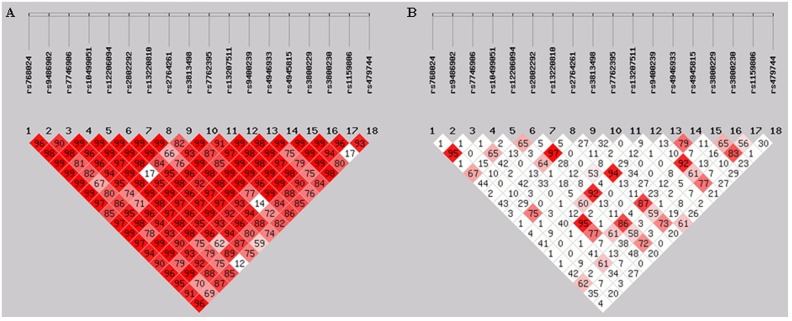
Linkage disequilibrium plot of the 18 *FOXO3* SNPs genotyped. Linkage disequilibrium was quantified as D' (A) and *r*^2^ (B), calculated in all subjects with the web tool SHEsis. Note: the darker the color, the higher the values.

Pairwise D' values between SNPs 5 and 9 (i.e. rs12206094 and rs3813498), SNPs 9 and 16 (i.e. rs3813498 and rs3800230), SNPs 5 and 18 (i.e. rs12206094 and rs479744), as well as SNPs 16 and 18 (i.e.rs3800230 and rs479744) were lower than 0.20, indicating that each of these pairwise SNPs were not in LD (Fig 1A). Other pairwise D' values between the 18 SNPs were greater than 0.59. Pairwise *r*^2^ values between SNPs 1 and 3 (i.e. rs768024 and rs7746906), SNPs 4 and 13 (i.e. rs10499051 and rs4946933), as well as SNPs 15 and 17 (i.e. rs3800229 and rs1159806) were higher than 0.80, suggesting that each of these pairwise SNPs were in high LD (*r*^2^ = 0.95, 0.95 and 0.83, respectively) (Fig 1B). SNPs 6, 8, 12 and 15 (i.e. rs2802292, rs2764261, rs9400239 and rs3800229) were also in high LD (all pairwise *r*^2^>0.85). Almost all (6/7) of the other pairwise *r*^2^ values were lower than 0.50.

We conducted haplotype association analyses using all SNPs in order to investigate their combined effect on human longevity (Table D in S1 File). Next, we performed haplotype analyses focusing on the SNPs significantly associated with male longevity. Among the five male-longevity-associated SNPs, rs10499051 and rs4946933 were in high LD (*r*^2^ = 0.95) (Fig 1B). Therefore, we performed haplotype analyses including only four male-longevity-associated SNPs (rs10499051, rs7762395, rs4945815 and rs3800230), not including rs4946933, and the results were similar to those got from haplotype analyses using all SNPs. For convenience and clarity of presentation, we only described the results produced by haplotype analyses using these four SNPs (Table 3).

**Table 3:** Haplotype analysis of male-longevity-associated SNPs in *FOXO3*.

Haplotype	rs10499051-rs7762395- rs4945815-rs3800230	Frequency		OR (95% CI)	*P*	*P*c
		LLIs	Controls			
All subjects						
1	A-G-C-T	0.663	0.664	1.00		
2	A-G-C-***G***	0.203	0.200	1.02 (0.85–1.24)	0.81	1
3	A-***A***-C-T	0.0587	0.0706	0.85 (0.63–1.14)	0.27	1
4	***G***-G-***T***-***G***	0.0633	0.0496	1.28 (0.93–1.76)	0.14	0.7
5	***G***-G-C-T	0.009	0.011	0.87 (0.39–1.92)	0.73	1
Rare haplotypes		0.00300	0.00500	0.51 (0.08–3.05)	0.46	1
Global					0.42	
Men						
1	A-G-C-T	0.628	0.673	1.00		
2	A-G-C-***G***	0.226	0.197	1.26 (0.82–1.95)	0.29	1
3	A-***A***-C-T	0.0196	0.0570	0.40 (0.13–1.22)	0.11	0.44
4	***G***-G-***T***-***G***	0.123	0.0566	2.36 (1.20–4.63)	**0.013**	0.052
Rare haplotypes		0.00490	0.0158	0.36 (0.04–3.19)	0.36	1
Global					**0.012**	
Women						
1	A-G-C-T	0.670	0.662	1.00		
2	A-G-C-***G***	0.198	0.201	0.98 (0.79–1.21)	0.85	1
3	A-***A***-C-T	0.0664	0.0737	0.90 (0.66–1.23)	0.5	1
4	***G***-G-***T***-***G***	0.0516	0.0480	1.06 (0.73–1.54)	0.75	1
5	***G***-G-C-T	0.0103	0.0113	0.94 (0.41–2.15)	0.89	1
Rare haplotypes		0.00330	0.00480	0.58 (0.10–3.49)	0.55	1
Global					0.95	

Significant results at *P*<0.05 are in bold. Alleles in bold and italic are different from the corresponding alleles in Haplotype 1 (A-G-C-T). *P*c, *P* values after Bonferroni correction using the number of haplotypes tested (n = 5, 4 and 5 for all subjects, men and women, respectively).

For both genders combined, no association was observed between haplotypes and human longevity (*P* = 0.42). When stratifying by gender, as expected, the ***G***-G-***T***-***G*** haplotype (minor allele for rs10499051, major allele for rs7762395, minor alleles for rs4945815 and rs3800230) was more prevalent in the male LLIs than in the male controls (OR = 2.36, 95% CI = 1.20–4.63, *P* = 0.013). However, this association did not withstand Bonferroni correction (*P*c = 0.052). The A-***A***-C-T haplotype (major allele for rs10499051, minor allele for rs7762395, major alleles for rs4945815 and rs3800230) was less frequent in male LLIs than in male controls but this did not reach statistical significance as expected (OR = 0.40, 95% CI = 0.13–1.22, *P* = 0.11). No nominally significant effects were found in Women.

The male LLIs had significantly higher frequency of the ***G***-G-***T***-***G*** haplotype (0.123 vs 0.0516, *P* = 0.0004) than the female LLIs whereas male and female controls had similar frequency of the ***G***-G-***T***-***G*** haplotype (0.0566 vs 0.0480, *P* = 0.64) (Table E in S1 File). Therefore, the association between the ***G***-G-***T***-***G*** haplotype and male longevity did not seem to be caused by a chance finding, although it did not withstand Bonferroni correction.

A small number of Li subjects (n = 107) were included in our study and thus we also restricted analyses to Han subjects. The results were similar to those in all subjects except for SNP rs3800230 (Tables F-K in S1 File). The minor allele of SNP rs3800230 exhibited a trend for association with male longevity, but this did not reach statistical significance (OR = 1.43, 95% CI = 0.96–2.14, *P* = 0.081) in the Chinese Han population (Table G in S1 File). However, just as the whole population, the male LLIs had significantly higher MAF of SNP rs3800230 (33.9% vs 25.0%, *P* = 0.012) as compared to the female LLIs whereas the male and female controls had similar MAF of it (26.5% vs 25.5%, *P* = 0.71) in the Chinese Han population (Table H in S1 File).

## Discussion

In this study, we studied on 18 common SNPs across the *FOXO3* to detect association with human longevity in a Chinese population. In initial analysis, allele and genotype distributions of each SNP tested did not differ between the LLIs and younger controls. After stratification by gender, five *FOXO3* intronic SNPs and one haplotype consisting of the alleles of four male-longevity-associated SNPs showed significant association with human longevity in men only. However, these associations did not retain statistical significance after Bonferroni correction. One interpretation is that these associations are due to chance and that the SNPs examined do not influence human lifespan. However, Bonferroni correction is generally overly stringent for genetic association analysis. The observed associations may be real, but need confirmation, for example by performing a study using a larger sample size.

Seven previous candidate gene studies have explored associations between variants in *FOXO3* and human longevity in Asians, six in Chinese and one in Japanese. Most of these studies investigated only a small number of SNPs, and their results have not been replicated. Our study analyzed 18 SNPs in *FOXO3*.

Willcox *et al*. (2008) [9] investigated three *FOXO3* SNPs (rs2764264, rs13217795 and rs2802292 (SNP 6)) in an ethnic Japanese population from the Island of Oahu, Hawaii, with 213 male LLIs aged >95 years and 402 male controls (mean age 78.5 years). They observed that all three SNPs were associated with male longevity. All three *FOXO3* SNPs are in high LD in Japanese in Tokyo (JPT) (*r*^2^ = 0.843–0.972) (available from the 1000 Genomes Project, based on GRCh38) [17]. However, in our study, SNP 6 was not associated with male longevity.

Li *et al*. (2009) [12] investigated three *FOXO3* SNPs (rs2253310, rs2802292 (SNP 6) and rs4946936) in 761 LLIs (mean age 102.3 years) and 1056 controls (mean age 47.1 years) from southern China and in 350 LLIs (mean age 100.8 years) and 350 controls (mean age 41.4 years) from northern China. They found that all three SNPs were associated with longevity. Two of the three *FOXO3* SNPs (rs2253310 and rs2802292 (SNP 6)) are almost in perfect LD in the CHB and CHS populations (*r*^2^ = 1 and 0.977, respectively) (available from the 1000 Genomes Project, based on GRCh38) [17]. However, in our study, SNP 6 and SNP 15 which is in high LD with rs4946936 in the CHB and CHS populations (*r*^2^ = 0.966 and 0.976, respectively) (available from the 1000 Genomes Project, based on GRCh38) [17] were not associated with longevity. Zeng *et al*. (2010) [18] reanalyzed the genotypic data in southern Chinese produced by Li *et al*. (2009)[12] and found that gene-gene interactions between *FOXO1* rs2755209 and *FOXO3* rs2253310 or *FOXO3* rs2802292 (SNP 6) decrease survival likelihood.

Li *et al*. (2010) [19] investigated eight *FOXO3* SNPs in 177 longevity subjects (aged≥90 years) and 148 controls (aged 48–89 years) from Bama County, Guangxi province and found that five SNPs (rs2764264, rs9400239 (SNP 12), rs13217795, rs2802288 and rs2802292 (SNP 6)) were associated with longevity (*P* = 0.032–0.038). Six of the eight *FOXO3* SNPs (i.e. the five associated SNPs and rs2802290) are in high LD in the CHB and CHS populations (all *r*^2^>0.85) (available from the 1000 Genomes Project, based on GRCh38) [17]. They also showed that rs7762395 (SNP 10) was not associated with longevity, but in our study, SNP 10 was associated with male longevity at the *P*<0.05 level. Possible drawbacks of Li *et al*. (2010) [19] are total absence of gender stratification and small sample size. Li *et al*. (2010) [19] and we both observed that SNP 7 was not associated with longevity.

He *et al*. (2014) [20] investigated two *FOXO3* SNPs (rs2153960 and rs13217795) in 567 longevity subjects (mean age 94.1 years) and 508 controls (mean age 51.7 years) from Dujianyan district of Sichuan region and found that rs13217795 was associated with human longevity. The two *FOXO3* SNPs are almost in perfect LD in the CHB and CHS populations (*r*^2^ = 1 and 0.977, respectively) (available from the 1000 Genomes Project, based on GRCh38) [17]. However, in our study, SNP 12 in perfect LD with rs13217795 in the CHB and CHS populations (*r*^2^ = 1) (available from the 1000 Genomes Project, based on GRCh38) [17] was not associated with human longevity.

Sun *et al*. (2015) [21] investigated seven *FOXO3* SNPs in 506 longevity subjects (mean age 92.5 years) and 830 controls (mean age 45.9 years) from the Red River Basin in South China and found that rs2802288*A and rs2802292*G were beneficial to longevity. Of note, rs2802288 and rs2802292 (SNP 6) are almost in perfect LD in the CHB and CHS populations (*r*^2^ = 1 and 0.977, respectively) (available from the 1000 Genomes Project, based on GRCh38) [17]. Five of the seven *FOXO3* SNPs (rs2802288, rs2802290, rs2802292 (SNP 6), rs2764264 and rs13217795) are in high LD in the CHB and CHS populations (all *r*^2^>0.85) (available from the 1000 Genomes Project, based on GRCh38) [17]. They also showed that SNP rs3800231 was associated with human longevity but did not remain significant after Bonferroni correction. In our study, SNP 15 in perfect LD with SNP rs3800231 in the CHB and CHS populations (available from the 1000 Genomes Project, based on GRCh38) [17] showed no association with human longevity.

Li *et al*. (2016) [22] investigated five *FOXO3* SNPs in 576 longevity subjects (mean age 92.8 years) and 626 controls (mean age 34.1 years) from Sichuan region and found that rs9486902 (SNP 2) was associated with female longevity while not in our study. Among the five SNPs, rs13217795 and rs9400239 (SNP 12) are in perfect LD in the CHB and CHS populations (*r*^2^ = 1) (available from the 1000 Genomes Project, based on GRCh38) [17]. They also showed SNP rs1935949 was not associated with human longevity. Similarly, in our study, SNP 15 in perfect LD with SNP rs1935949 in the CHB and CHS populations (available from the 1000 Genomes Project, based on GRCh38) [17] was not associated with human longevity.

It should be noted the above studies conducted in Asians did not investigate the five SNPs we have identified associated with male longevity at *P*<0.05, or any SNPs in high LD with the five SNPs, except for the study of Li *et al*. (2010) [19] which included one (SNP 10). In our study, the number of SNPs is obviously much more and the SNPs investigated are more representative although a few SNPs are in high LD. In addition, among the above studies conducted in Asians, Li *et al*. (2009) [12] and we recruited older subjects (mean age>100 years) as cases. According to the sixth Chinese census database in 2010, among China's 31 provinces, autonomous regions and municipalities, the highest number of centenarians per 10,000 inhabitants ≥ 65 years of age is in Hainan (16.64), followed by Guangxi (7.00), Guangdong (6.07), Xinjiang (5.25), Shanghai (3.98) [23]. This enabled us to recruit more LLIs from Hainan into our study.

Among the candidate gene studies conducted in Asians, Li *et al*. (2009) [12] and Li *et al*. (2016) [22] also performed gender-stratified analyses. Li *et al*. (2009) [12] did not observed gender-dependent effects of *FOXO3* variants on human longevity while Li *et al*. (2016) [22] observed gender-dependent effect of rs9486902 (SNP 2) on human longevity. The two studies did not include the five SNPs we have identified to be associated with male longevity at *P*<0.05, or any SNPs in high LD with the five SNPs.

Recently, Zeng *et al*. (2016) [24] performed a genome-wide association study using the Illumina HumanOmniZhongHua-8 BeadChips in the Han Chinese population with 2,178 centenarians and 2,299 mid-age controls. They also found 4 *FOXO3* SNPs are associated with longevity with a *P* < 0.04.

There are several studies comprehensively evaluating common variation in *FOXO3* for association with human lifespan in Caucasians [10, 11, 13, 25]. These studies reported 5 of 16 tagging SNPs (four specific to males and one to females) in Italians in the absence of multiple testing correction [10], 3 of 16 tagging SNPs (both genders) in Germans [11], 2 of 16 tagging SNPs (specific to females) in Americans (northern and western European ancestry) [25], and 8 of 15 tagging SNPs (specific to males) in Danes to be associated with longevity [13].

CHB and CHS show quite distinct patterns of LD from Utah residents with northern European ancestry (CEU). For example, SNP 4 (rs10499051) captures rs138885215 and rs1408769 in CEU with *r*^2^ = 1 but not in CHB and CHS (between SNP 4 and rs138885215: *r*^2^ = 0.522 and 0.467, respectively; between SNP 4 and rs1408769: *r*^2^ = 0.522 and 0.537, respectively) whereas captures rs57099886, rs202242855, rs72940678, rs72940694 and rs12055603 in CHB and CHS with *r*^2^ = 1 but not in CEU (all pairwise *r*^2^ between SNP 4 and these 5 SNPs are 0.421) (available from the 1000 Genomes Project, based on GRCh38) [17]. Different LD and haplotype patterns between populations could lead to some disparities of longevity association between different populations. Studies in Chinese will help reveal the disparities and provide a better understanding of the genetic basis of human longevity. Therefore, in this study, we comprehensively evaluated common variants in *FOXO3* for associations with human lifespan in a Chinese population.

Just like our study, most of the various reported longevity-associated SNPs are located in or near intron 2 of the 125-kb *FOXO3* gene [1, 26, 27]. A meta-analysis of the studies published prior to July 2013 found that 4 SNPs in intron 2 and 1 in intron 1 of the 125-kb *FOXO3* gene retained statistically significant associations with longevity [28]. Of the five SNPs associated with male longevity at *P*<0.05 in our study, SNP 4, 10 and 13 are in intron 2 while SNP 14 and 16 are in intron 3. A study with extensive sequence analyses of coding DNA suggested that coding variants of the *FOXO3* gene may not be key players in human aging and the functional SNPs are most likely noncoding SNPs [27]. The causal SNP(s) and the molecular mechanism underlying the effect of the longevity-associated allele(s) on human longevity remain to be clarified.

Of the five SNPs associated with male longevity at *P*<0.05 in our study, SNP 14 (rs4945815) is monomorphic in CEU (available from the 1000 Genomes Project, based on GRCh38) [17]. SNP 14 and any SNPs in high LD with it in CHB and CHS have not been investigated previously for human longevity. We first investigated and demonstrated that SNP 14 was associated with male longevity at *P*<0.05. Among the significant associations for the five SNPs, *P* value for SNP 14 was smallest. And the MAF of SNP rs4945815 in male LLIs was approximately 2.37 times as much as that in female LLIs.

In our study, SNP 4 (rs10499051) and SNP 13 (rs4946933) were in high LD (*r*^2^ = 0.95) and both associated with male longevity. SNP 4 was also associated with longevity only for men in Italians [10], while only for women in Danes [13]. But none of them remained significant when correcting by Bonferroni correction.

SNP 10 (rs7762395) was associated with longevity for men in our study and in Danes [13] whereas, in Germans, for both genders combined [11]. Both of the associations in Danes and Germans passed Bonferroni correction. In 2014, a meta-analysis performed in four independent studies showed absence of association between SNP 10 and longevity [28]. However, later, SNP 10 was found to be significantly associated with longevity [29, 30] and bone fracture [31], one of aging phenotypes, in Caucasians.

SNP 16 (rs3800230) is in perfect or very high LD with rs3800232 in the CEU, CHB and CHS populations (*r*^2^ = 1, 1 and 0.971, respectively) (available from the 1000 Genomes Project, based on GRCh38) [17]. SNP rs3800232 was significant only for the male centenarians in Danes but did not remain after Bonferroni correction [13]. Similarly, our study showed that SNP 16 was associated with longevity only in men although it lost the significance after Bonferroni correction.

A gender-dependent effect of the *FOXO3* gene polymorphisms on human longevity was reported by previous studies [10, 13, 22, 25, 28]. Our study, again, confirmed *FOXO3* may exert effects on human longevity in a sex-dependent manner. Women live longer than men. Men and women seem to follow different strategies to reach longevity. One of mechanisms of women’s longevity advantage is hormonal influences on inflammatory and immunological responses, or greater resistance to oxidative damage. Hormones may differentially affect gene expression, thus leading to the gender-specific susceptibility to longevity and the genetic contribution to longevity may be affected by gender [32–34].

Although the sample size of our study was relatively large among the published case-control genetic association studies in human longevity, it was moderate for genetic association studies, especially in men. Our study only included 102 long-lived men and 159 younger men. Therefore, the sample size may limit the power of our study to detect association, especially in men. More LLIs, especially long-lived men are required in further studies.

In summary, we performed a comprehensive study to investigate whether the *FOXO3* common variants are associated with human longevity in a Chinese population and identified one new variant, as well as confirming four previously reported variants associated with human longevity in men only. These associations were nominally significant before Bonferroni correction. Bonferroni correction is too conservative for association studies. Therefore, there is nevertheless a strong possibility that the effects of these nominally significant SNPs on human longevity will be confirmed in other populations. The causal variant(s) and the molecular mechanisms underlying the association with longevity need to be addressed in future studies.

## Supporting Information

S1 File Table A: Genotype and allele frequencies of *FOXO3* polymorphisms in the long-lived individuals and controls. Table B: Genotype and allele frequencies of *FOXO3* polymorphisms in the long-lived individuals and controls when stratifying by gender. Table C: Allele frequencies of the five male-longevity-associated *FOXO3* polymorphisms in the long-lived individuals and controls. Table D: Association of *FOXO3* haplotypes with human longevity. Table E: Haplotype frequencies of male-longevity-associated SNPs in *FOXO3* in the long-lived individuals and controls. Table F: Genotype and allele frequencies of *FOXO3* polymorphisms in the Chinese Han long-lived individuals and controls. Table G: Genotype and allele frequencies of *FOXO3* polymorphisms in the Chinese Han long-lived individuals and controls when stratifying by gender. Table H: Allele frequencies of the five male-longevity-associated *FOXO3* polymorphisms in the Chinese Han long-lived individuals and controls. Table I: Association of *FOXO3* haplotypes with human longevity in the Chinese Han population. Table J: Haplotype analysis of male-longevity-associated SNPs in *FOXO3* in the Chinese Han population. Table K: Haplotype frequencies of male-longevity-associated SNPs in *FOXO3* in the Chinese Han long-lived individuals and controls.
(DOC)
