# Supplementary material for: Genetic Association Analysis of Common Variants in FOXO3 Related to Longevity in a Chinese Population
Source: PLoS One. 2016 Dec 9;11(12):e0167918. doi: 10.1371/journal.pone.0167918 (PMC5148017; doi:10.1371/journal.pone.0167918)
Supplement: S1 File — Table A: Genotype and allele frequencies of FOXO3 polymorphisms in the long-lived individuals and controls. Table B: Genotype and allele frequencies of FOXO3 polymorphisms in the long-lived individuals and controls when stratifying by gender. Table C: Allele frequencies of the five male-longevity-associated FOXO3 polymorphisms in the long-lived individuals and controls. Table D: Association of FOXO3 haplotypes with human longevity. Table E: Haplotype frequencies of male-longevity-associated SNPs in FOXO3 in the long-lived individuals and controls. Table F: Genotype and allele frequencies of FOXO3 polymorphisms in the Chinese Han long-lived individuals and controls. Table G: Genotype and allele frequencies of FOXO3 polymorphisms in the Chinese Han long-lived individuals and controls when stratifying by gender. Table H: Allele frequencies of the five male-longevity-associated FOXO3 polymorphisms in the Chinese Han long-lived individuals and controls. Table I: Association of FOXO3 haplotypes with human longevity in the Chinese Han population. Table J: Haplotype analysis of male-longevity-associated SNPs in FOXO3 in the Chinese Han population. Table K: Haplotype frequencies of male-longevity-associated SNPs in FOXO3 in the Chinese Han long-lived individuals and controls. (DOC) [file pone.0167918.s001.doc]

| **Table A. Genotype and allele frequencies of *FOXO3* polymorphisms in the long-lived individuals and controls.** | | | | | |
| --- | --- | --- | --- | --- | --- |
| dbSNP ID | Genotype/Allele | LLIs | Controls | OR(95%CI) | *P* |
| rs768024 | G/G | 395 (64.1%) | 546 (64.5%) | 1.00 | 0.09 |
|  | G/A | 191 (31%) | 277 (32.7%) | 0.95 (0.76-1.19) |
|  | A/A | 30 (4.9%) | 23 (2.7%) | **1.80 (1.03-3.15)** |
|  | G | 981 (79.6%) | 1369 (80.9%) | 1.00 |  |
|  | A | 251 (20.4%) | 323 (19.1%) | 1.09 (0.90-1.31) | 0.39 |
| rs9486902 | C/C | 520 (85.2%) | 713 (85%) | 1.00 | 0.73 |
|  | C/T | 87 (14.3%) | 119 (14.2%) | 1.00 (0.74-1.35) |
|  | T/T | 3 (0.5%) | 7 (0.8%) | 0.59 (0.15-2.28) |
|  | C | 1127 (92.4%) | 1545 (92.1%) | 1.00 |  |
|  | T | 93 (7.6%) | 133 (7.9%) | 0.96 (0.73-1.26) | 0.76 |
| rs7746906 | G/G | 381 (62.2%) | 545 (64.4%) | 1.00 | 0.09 |
|  | G/A | 201 (32.8%) | 278 (32.9%) | 1.03 (0.83-1.29) |
|  | A/A | 30 (4.9%) | 23 (2.7%) | **1.87 (1.07-3.26)** |
|  | G | 963 (78.7%) | 1368 (80.9%) | 1.00 |  |
|  | A | 261 (21.3%) | 324 (19.1%) | 1.15 (0.95-1.38) | 0.14 |
| rs10499051 | A/A | 526 (85.4%) | 743 (87.8%) | 1.00 | 0.35 |
|  | G/A | 87 (14.1%) | 98 (11.6%) | 1.25 (0.92-1.71) |
|  | G/G | 3 (0.5%) | 5 (0.6%) | 0.85 (0.20-3.56) |
|  | A | 1139 (92.5%) | 1584 (93.6%) | 1.00 |  |
|  | G | 93 (7.5%) | 108 (6.4%) | 1.20 (0.90-1.59) | 0.22 |
| rs12206094 | C/C | 343 (55.8%) | 459 (54.3%) | 1.00 | 0.29 |
|  | C/T | 220 (35.8%) | 329 (38.9%) | 0.89 (0.72-1.12) |
|  | T/T | 52 (8.5%) | 57 (6.8%) | 1.22 (0.82-1.82) |
|  | C | 906 (73.7%) | 1247 (73.8%) | 1.00 |  |
|  | T | 324 (26.3%) | 443 (26.2%) | 1.01 (0.85-1.19) | 0.94 |
| rs2802292 | T/T | 246 (40.5%) | 353 (42.1%) | 1.00 | 0.82 |
|  | G/T | 288 (47.4%) | 388 (46.2%) | 1.07 (0.85-1.33) |
|  | G/G | 74 (12.2%) | 98 (11.7%) | 1.08 (0.77-1.53) |
|  | T | 780 (64.1%) | 1094 (65.2%) | 1.00 |  |
|  | G | 436 (35.9%) | 584 (34.8%) | 1.05 (0.90-1.23) | 0.55 |
| rs13220810 | T/T | 507 (82.3%) | 712 (84.3%) | 1.00 | 0.27 |
|  | C/T | 102 (16.6%) | 129 (15.3%) | 1.11 (0.84-1.47) |
|  | C/C | 7 (1.1%) | 4 (0.5%) | 2.46 (0.72-8.44) |
|  | T | 1116 (90.6%) | 1553 (91.9%) | 1.00 |  |
|  | C | 116 (9.4%) | 137 (8.1%) | 1.18 (0.91-1.53) | 0.22 |
| rs2764261 | G/G | 255 (41.4%) | 357 (42.2%) | 1.00 | 0.95 |
|  | G/A | 289 (46.9%) | 392 (46.3%) | 1.03 (0.83-1.29) |
|  | A/A | 72 (11.7%) | 97 (11.5%) | 1.04 (0.74-1.47) |
|  | G | 799 (64.9%) | 1106 (65.4%) | 1.00 |  |
|  | A | 433 (35.1%) | 586 (34.6%) | 1.02 (0.88-1.20) | 0.77 |
| rs3813498 | T/T | 400 (65.2%) | 578 (68.5%) | 1.00 | 0.34 |
|  | C/T | 196 (32%) | 240 (28.4%) | 1.18 (0.94-1.48) |
|  | C/C | 17 (2.8%) | 26 (3.1%) | 0.94 (0.51-1.76) |
|  | T | 996 (81.2%) | 1396 (82.7%) | 1.00 |  |
|  | C | 230 (18.8%) | 292 (17.3%) | 1.11 (0.91-1.34) | 0.31 |
| rs7762395 | G/G | 548 (89%) | 732 (86.5%) | 1.00 | 0.36 |
|  | G/A | 63 (10.2%) | 107 (12.7%) | 0.79 (0.57-1.09) |
|  | A/A | 5 (0.8%) | 7 (0.8%) | 0.95 (0.30-3.02) |
|  | G | 1159 (94.1%) | 1571 (92.8%) | 1.00 |  |
|  | A | 73 (5.9%) | 121 (7.2%) | 0.83 (0.62-1.11) | 0.2 |
| rs13207511 | A/A | 439 (71.3%) | 632 (74.7%) | 1.00 | 0.33 |
|  | G/A | 165 (26.8%) | 201 (23.8%) | 1.18 (0.93-1.50) |
|  | G/G | 12 (2%) | 13 (1.5%) | 1.33 (0.60-2.94) |
|  | A | 1043 (84.7%) | 1465 (86.6%) | 1.00 |  |
|  | G | 189 (15.3%) | 227 (13.4%) | 1.17 (0.95-1.45) | 0.14 |
| rs9400239 | C/C | 255 (41.4%) | 347 (41%) | 1.00 | 0.99 |
|  | C/T | 286 (46.4%) | 396 (46.8%) | 0.98 (0.79-1.23) |
|  | T/T | 75 (12.2%) | 103 (12.2%) | 0.99 (0.71-1.39) |
|  | C | 796 (64.6%) | 1090 (64.4%) | 1.00 |  |
|  | T | 436 (35.4%) | 602 (35.6%) | 0.99 (0.85-1.16) | 0.92 |
| rs4946933 | G/G | 526 (85.5%) | 738 (87.3%) | 1.00 | 0.46 |
|  | G/A | 86 (14%) | 101 (11.9%) | 1.19 (0.88-1.63) |
|  | A/A | 3 (0.5%) | 6 (0.7%) | 0.70 (0.17-2.82) |
|  | G | 1138 (92.5%) | 1577 (93.3%) | 1.00 |  |
|  | A | 92 (7.5%) | 113 (6.7%) | 1.13 (0.85-1.49) | 0.41 |
| rs4945815 | C/C | 539 (87.6%) | 763 (90.2%) | 1.00 | 0.24 |
|  | C/T | 74 (12%) | 79 (9.3%) | 1.33 (0.95-1.85) |
|  | T/T | 2 (0.3%) | 4 (0.5%) | 0.71 (0.13-3.88) |
|  | C | 1152 (93.7%) | 1605 (94.9%) | 1.00 |  |
|  | T | 78 (6.3%) | 87 (5.1%) | 1.24 (0.91-1.70) | 0.17 |
| rs3800229 | T/T | 258 (42.1%) | 352 (41.8%) | 1.00 | 0.99 |
|  | G/T | 283 (46.2%) | 390 (46.3%) | 0.99 (0.79-1.24) |
|  | G/G | 72 (11.8%) | 100 (11.9%) | 0.98 (0.70-1.38) |
|  | T | 799 (65.2%) | 1094 (65.0%) | 1.00 |  |
|  | G | 427 (34.8%) | 590 (35.0%) | 0.99 (0.85-1.16) | 0.91 |
| rs3800230 | T/T | 328 (53.2%) | 465 (55%) | 1.00 | 0.59 |
|  | G/T | 244 (39.6%) | 331 (39.1%) | 1.05 (0.84-1.30) |
|  | G/G | 44 (7.1%) | 50 (5.9%) | 1.25 (0.81-1.92) |
|  | T | 900 (73.1%) | 1261 (74.5%) | 1.00 |  |
|  | G | 332 (26.9%) | 431 (25.5%) | 1.08 (0.91-1.28) | 0.37 |
| rs1159806 | A/A | 243 (39.5%) | 341 (40.3%) | 1.00 | 0.94 |
|  | A/T | 292 (47.4%) | 394 (46.6%) | 1.04 (0.83-1.30) |
|  | T/T | 81 (13.2%) | 111 (13.1%) | 1.02 (0.74-1.42) |
|  | A | 778 (63.1%) | 1076 (63.6%) | 1.00 |  |
|  | T | 454 (36.9%) | 616 (36.4%) | 1.02 (0.87-1.19) | 0.8 |
| rs479744 | G/G | 426 (69.3%) | 590 (69.8%) | 1.00 | 0.73 |
|  | G/T | 175 (28.5%) | 231 (27.3%) | 1.05 (0.83-1.32) |
|  | T/T | 14 (2.3%) | 24 (2.8%) | 0.81 (0.41-1.58) |
|  | G | 1027 (83.5%) | 1411 (83.5%) | 1.00 |  |
|  | T | 203 (16.5%) | 279 (16.5%) | 1.00 (0.82-1.22) | 1 |

| **Table B. Genotype and allele frequencies of *FOXO3* polymorphisms in the long-lived individuals and controls when stratifying by gender.** | | | | | |
| --- | --- | --- | --- | --- | --- |
| dbSNP ID | Genotype/Allele | LLIs | Controls | OR(95%CI) | *P* |
| Men |  |  |  |  |  |
| rs768024 | G/G | 64 (62.8%) | 103 (64.8%) | 1.00 | 0.14 |
|  | G/A | 29 (28.4%) | 51 (32.1%) | 0.92 (0.53-1.59) |
|  | A/A | 9 (8.8%) | 5 (3.1%) | 2.90 (0.93-9.03) |
|  | G | 157 (77.0%) | 257 (80.8%) | 1.00 |  |
|  | A | 47 (23.0%) | 61 (19.2%) | 1.24 (0.82-1.89) | 0.31 |
| rs9486902 | C/C | 93 (91.2%) | 139 (87.4%) | 1.00 | 0.43 |
|  | C/T | 9 (8.8%) | 19 (11.9%) | 0.71 (0.31-1.63) |
|  | T/T | 0 (0%) | 1 (0.6%) |  |
|  | C | 195 (95.6%) | 297 (93.4%) | 1.00 |  |
|  | T | 9 (4.4%) | 21 (6.6%) | 0.65 (0.29-1.46) | 0.29 |
| rs7746906 | G/G | 60 (60%) | 104 (65.4%) | 1.00 | 0.13 |
|  | G/A | 31 (31%) | 50 (31.4%) | 1.07 (0.62-1.86) |
|  | A/A | 9 (9%) | 5 (3.1%) | 3.12 (1.00-9.74) |
|  | G | 151 (75.5%) | 258 (81.1%) | 1.00 |  |
|  | A | 49 (24.5%) | 60 (18.9%) | 1.37 (0.90-2.08) | 0.14 |
| rs10499051 | A/A | 77 (75.5%) | 136 (85.5%) | 1.00 | 0.067 |
|  | G/A | 24 (23.5%) | 23 (14.5%) | 1.84 (0.98-3.48) |
|  | G/G | 1 (1%) | 0 (0%) |  |
|  | A | 178 (87.3%) | 295 (92.8%) | 1.00 |  |
|  | G | 26 (12.7%) | 23 (7.2%) | **1.96 (1.06-3.62)** | **0.032** |
| rs12206094 | C/C | 60 (58.8%) | 90 (56.6%) | 1.00 | 0.56 |
|  | C/T | 32 (31.4%) | 58 (36.5%) | 0.83 (0.48-1.42) |
|  | T/T | 10 (9.8%) | 11 (6.9%) | 1.36 (0.55-3.41) |
|  | C | 152 (74.5%) | 238 (74.8%) | 1.00 |  |
|  | T | 52 (25.5%) | 80 (25.2%) | 1.02 (0.69-1.50) | 0.93 |
| rs2802292 | T/T | 37 (36.3%) | 67 (42.1%) | 1.00 | 0.42 |
|  | G/T | 49 (48%) | 75 (47.2%) | 1.18 (0.69-2.03) |
|  | G/G | 16 (15.7%) | 17 (10.7%) | 1.70 (0.77-3.76) |
|  | T | 123 (60.3%) | 209 (65.7%) | 1.00 |  |
|  | G | 81 (39.7%) | 109 (34.3%) | 1.27 (0.88-1.84) | 0.2 |
| rs13220810 | T/T | 86 (84.3%) | 130 (81.8%) | 1.00 | 0.56 |
|  | C/T | 16 (15.7%) | 28 (17.6%) | 0.86 (0.44-1.69) |
|  | C/C | 0 (0%) | 1 (0.6%) |  |
|  | T | 188 (92.2%) | 288 (90.6%) | 1.00 |  |
|  | C | 16 (7.8%) | 30 (9.4%) | 0.81 (0.42-1.55) | 0.52 |
| rs2764261 | G/G | 37 (36.3%) | 68 (42.8%) | 1.00 | 0.51 |
|  | G/A | 50 (49%) | 73 (45.9%) | 1.26 (0.73-2.16) |
|  | A/A | 15 (14.7%) | 18 (11.3%) | 1.53 (0.69-3.39) |
|  | G | 124 (60.8%) | 209 (65.7%) | 1.00 |  |
|  | A | 80 (39.2%) | 109 (34.3%) | 1.24 (0.86-1.80) | 0.25 |
| rs3813498 | T/T | 66 (64.7%) | 112 (70.4%) | 1.00 | 0.62 |
|  | C/T | 34 (33.3%) | 44 (27.7%) | 1.31 (0.76-2.25) |
|  | C/C | 2 (2%) | 3 (1.9%) | 1.13 (0.18-6.95) |
|  | T | 166 (81.4%) | 268 (84.3%) | 1.00 |  |
|  | C | 38 (18.6%) | 50 (15.7%) | 1.25 (0.77-2.02) | 0.37 |
| rs7762395 | G/G | 98 (96.1%) | 141 (88.7%) | 1.00 | 0.071 |
|  | G/A | 4 (3.9%) | 17 (10.7%) | 0.34 (0.11-1.04) |
|  | A/A | 0 (0%) | 1 (0.6%) |  |
|  | G | 200 (98.0%) | 299 (94.0%) | 1.00 |  |
|  | A | 4 (2.0%) | 19 (6.0%) | **0.32 (0.11-0.96)** | **0.024** |
| rs13207511 | A/A | 73 (71.6%) | 113 (71.1%) | 1.00 | 0.94 |
|  | G/A | 28 (27.4%) | 45 (28.3%) | 0.96 (0.55-1.68) |
|  | G/G | 1 (1%) | 1 (0.6%) | 1.55 (0.10-25.14) |
|  | A | 174 (85.3%) | 271 (85.2%) | 1.00 |  |
|  | G | 30 (14.7%) | 47 (14.8%) | 0.99 (0.59-1.68) | 0.98 |
| rs9400239 | C/C | 38 (37.2%) | 66 (41.5%) | 1.00 | 0.63 |
|  | C/T | 48 (47.1%) | 74 (46.5%) | 1.13 (0.66-1.93) |
|  | T/T | 16 (15.7%) | 19 (11.9%) | 1.46 (0.67-3.18) |
|  | C | 124 (60.8%) | 206 (64.8%) | 1.00 |  |
|  | T | 80 (39.2%) | 112 (35.2%) | 1.19 (0.82-1.71) | 0.35 |
| rs4946933 | G/G | 77 (75.5%) | 136 (85.5%) | 1.00 | 0.067 |
|  | G/A | 24 (23.5%) | 23 (14.5%) | 1.84 (0.98-3.48) |
|  | A/A | 1 (1%) | 0 (0%) |  |
|  | G | 178 (87.3%) | 295 (92.8%) | 1.00 |  |
|  | A | 26 (12.7%) | 23 (7.2%) | **1.96 (1.06-3.62)** | **0.032** |
| rs4945815 | C/C | 78 (76.5%) | 141 (88.7%) | 1.00 | **0.02** |
|  | C/T | 23 (22.5%) | 18 (11.3%) | **2.31 (1.17-4.54)** |
|  | T/T | 1 (1%) | 0 (0%) |  |
|  | C | 179 (87.7%) | 300 (94.3%) | 1.00 |  |
|  | T | 25 (12.3%) | 18 (5.7%) | **2.43 (1.26-4.68)** | **0.007** |
| rs3800229 | T/T | 38 (37.2%) | 66 (41.5%) | 1.00 | 0.45 |
|  | G/T | 47 (46.1%) | 75 (47.2%) | 1.09 (0.63-1.87) |
|  | G/G | 17 (16.7%) | 18 (11.3%) | 1.64 (0.76-3.56) |
|  | T | 123 (60.3%) | 207 (65.1%) | 1.00 |  |
|  | G | 81 (39.7%) | 111 (34.9%) | 1.23 (0.85-1.77) | 0.27 |
| rs3800230 | T/T | 43 (42.2%) | 86 (54.1%) | 1.00 | 0.077 |
|  | G/T | 46 (45.1%) | 63 (39.6%) | 1.46 (0.86-2.48) |
|  | G/G | 13 (12.8%) | 10 (6.3%) | **2.60 (1.05-6.41)** |
|  | T | 132 (64.7%) | 235 (73.9%) | 1.00 |  |
|  | G | 72 (35.3%) | 83 (26.1%) | **1.55 (1.05-2.28)** | **0.025** |
| rs1159806 | A/A | 37 (36.3%) | 66 (41.5%) | 1.00 | 0.4 |
|  | A/T | 47 (46.1%) | 74 (46.5%) | 1.13 (0.66-1.95) |
|  | T/T | 18 (17.6%) | 19 (11.9%) | 1.69 (0.79-3.61) |
|  | A | 121 (59.3%) | 206 (64.8%) | 1.00 |  |
|  | T | 83 (40.7%) | 112 (35.2%) | 1.26 (0.88-1.81) | 0.21 |
| rs479744 | G/G | 69 (67.7%) | 111 (69.8%) | 1.00 | 0.87 |
|  | G/T | 31 (30.4%) | 46 (28.9%) | 1.08 (0.63-1.87) |
|  | T/T | 2 (2%) | 2 (1.3%) | 1.61 (0.22-11.69) |
|  | G | 169 (82.8%) | 268 (84.3%) | 1.00 |  |
|  | T | 35 (17.2%) | 50 (15.7%) | 1.12 (0.68-1.84) | 0.65 |
| Women |  |  |  |  |  |
| rs768024 | G/G | 331 (64.4%) | 443 (64.5%) | 1.00 | 0.35 |
|  | G/A | 162 (31.5%) | 226 (32.9%) | 0.96 (0.75-1.23) |
|  | A/A | 21 (4.1%) | 18 (2.6%) | 1.56 (0.82-2.98) |
|  | G | 824 (80.2%) | 1112 (80.9%) | 1.00 |  |
|  | A | 204 (19.8%) | 262 (19.1%) | 1.05 (0.86-1.30) | 0.63 |
| rs9486902 | C/C | 427 (84.1%) | 574 (84.4%) | 1.00 | 0.81 |
|  | C/T | 78 (15.3%) | 100 (14.7%) | 1.05 (0.76-1.45) |
|  | T/T | 3 (0.6%) | 6 (0.9%) | 0.67 (0.17-2.70) |
|  | C | 932 (91.7%) | 1248 (91.8%) | 1.00 |  |
|  | T | 84 (8.3%) | 112 (8.2%) | 1.00 (0.75-1.35) | 0.98 |
| rs7746906 | G/G | 321 (62.7%) | 441 (64.2%) | 1.00 | 0.36 |
|  | G/A | 170 (33.2%) | 228 (33.2%) | 1.02 (0.80-1.31) |
|  | A/A | 21 (4.1%) | 18 (2.6%) | 1.60 (0.84-3.06) |
|  | G | 812 (79.3%) | 1110 (80.8%) | 1.00 |  |
|  | A | 212 (20.7%) | 264 (19.2%) | 1.10 (0.90-1.36) | 0.36 |
| rs10499051 | A/A | 449 (87.3%) | 607 (88.4%) | 1.00 | 0.58 |
|  | G/A | 63 (12.3%) | 75 (10.9%) | 1.14 (0.80-1.62) |
|  | G/G | 2 (0.4%) | 5 (0.7%) | 0.54 (0.10-2.80) |
|  | A | 961 (93.5%) | 1289 (93.8%) | 1.00 |  |
|  | G | 67 (6.5%) | 85 (6.2%) | 1.06 (0.76-1.46) | 0.75 |
| rs12206094 | C/C | 283 (55.2%) | 369 (53.8%) | 1.00 | 0.45 |
|  | C/T | 188 (36.6%) | 271 (39.5%) | 0.90 (0.71-1.15) |
|  | T/T | 42 (8.2%) | 46 (6.7%) | 1.19 (0.76-1.86) |
|  | C | 754 (73.5%) | 1009 (73.5%) | 1.00 |  |
|  | T | 272 (26.5%) | 363 (26.5%) | 1.00 (0.84-1.20) | 0.98 |
| rs2802292 | T/T | 209 (41.3%) | 286 (42.1%) | 1.00 | 0.91 |
|  | G/T | 239 (47.2%) | 313 (46%) | 1.04 (0.82-1.34) |
|  | G/G | 58 (11.5%) | 81 (11.9%) | 0.98 (0.67-1.43) |
|  | T | 657 (64.9%) | 885 (65.1%) | 1.00 |  |
|  | G | 355 (35.1%) | 475 (34.9%) | 1.01 (0.85-1.20) | 0.94 |
| rs13220810 | T/T | 421 (81.9%) | 582 (84.8%) | 1.00 | 0.13 |
|  | C/T | 86 (16.7%) | 101 (14.7%) | 1.18 (0.86-1.61) |
|  | C/C | 7 (1.4%) | 3 (0.4%) | 3.23 (0.83-12.55) |
|  | T | 928 (90.3%) | 1265 (92.2%) | 1.00 |  |
|  | C | 100 (9.7%) | 107 (7.8%) | 1.27 (0.96-1.69) | 0.099 |
| rs2764261 | G/G | 218 (42.4%) | 289 (42.1%) | 1.00 | 0.97 |
|  | G/A | 239 (46.5%) | 319 (46.4%) | 0.99 (0.78-1.27) |
|  | A/A | 57 (11.1%) | 79 (11.5%) | 0.96 (0.65-1.40) |
|  | G | 675 (65.7%) | 897 (65.3%) | 1.00 |  |
|  | A | 353 (34.3%) | 477 (34.7%) | 0.98 (0.83-1.17) | 0.85 |
| rs3813498 | T/T | 334 (65.4%) | 466 (68%) | 1.00 | 0.5 |
|  | C/T | 162 (31.7%) | 196 (28.6%) | 1.15 (0.90-1.48) |
|  | C/C | 15 (2.9%) | 23 (3.4%) | 0.91 (0.47-1.77) |
|  | T | 830 (81.2%) | 1128 (82.3%) | 1.00 |  |
|  | C | 192 (18.8%) | 242 (17.7%) | 1.08 (0.87-1.33) | 0.48 |
| rs7762395 | G/G | 450 (87.5%) | 591 (86%) | 1.00 | 0.69 |
|  | G/A | 59 (11.5%) | 90 (13.1%) | 0.86 (0.61-1.22) |
|  | A/A | 5 (1%) | 6 (0.9%) | 1.09 (0.33-3.61) |
|  | G | 959 (93.3%) | 1272 (92.6%) | 1.00 |  |
|  | A | 69 (6.7%) | 102 (7.4%) | 0.90 (0.66-1.23) | 0.51 |
| rs13207511 | A/A | 366 (71.2%) | 519 (75.5%) | 1.00 | 0.24 |
|  | G/A | 137 (26.6%) | 156 (22.7%) | 1.25 (0.95-1.62) |
|  | G/G | 11 (2.1%) | 12 (1.8%) | 1.30 (0.57-2.98) |
|  | A | 869 (84.5%) | 1194 (86.9%) | 1.00 |  |
|  | G | 159 (15.5%) | 180 (13.1%) | 1.22 (0.96-1.53) | 0.099 |
| rs9400239 | C/C | 217 (42.2%) | 281 (40.9%) | 1.00 | 0.87 |
|  | C/T | 238 (46.3%) | 322 (46.9%) | 0.96 (0.75-1.22) |
|  | T/T | 59 (11.5%) | 84 (12.2%) | 0.91 (0.62-1.33) |
|  | C | 672 (65.4%) | 884 (64.3%) | 1.00 |  |
|  | T | 356 (34.6%) | 490 (35.7%) | 0.95 (0.80-1.13) | 0.6 |
| rs4946933 | G/G | 449 (87.5%) | 602 (87.8%) | 1.00 | 0.54 |
|  | G/A | 62 (12.1%) | 78 (11.4%) | 1.07 (0.75-1.52) |
|  | A/A | 2 (0.4%) | 6 (0.9%) | 0.45 (0.09-2.22) |
|  | G | 960 (93.6%) | 1282 (93.4%) | 1.00 |  |
|  | A | 66 (6.4%) | 90 (6.6%) | 0.98 (0.71-1.35) | 0.9 |
| rs4945815 | C/C | 461 (89.9%) | 622 (90.5%) | 1.00 | 0.47 |
|  | C/T | 51 (9.9%) | 61 (8.9%) | 1.13 (0.76-1.67) |
|  | T/T | 1 (0.2%) | 4 (0.6%) | 0.34 (0.04-3.03) |
|  | C | 973 (94.8%) | 1305 (95.0%) | 1.00 |  |
|  | T | 53 (5.2%) | 69 (5.0%) | 1.03 (0.72-1.48) | 0.88 |
| rs3800229 | T/T | 220 (43%) | 286 (41.9%) | 1.00 | 0.78 |
|  | G/T | 236 (46.2%) | 315 (46.1%) | 0.97 (0.76-1.24) |
|  | G/G | 55 (10.8%) | 82 (12%) | 0.87 (0.59-1.28) |
|  | T | 676 (66.1%) | 887 (64.9%) | 1.00 |  |
|  | G | 346 (33.9%) | 479 (35.1%) | 0.95 (0.80-1.12) | 0.53 |
| rs3800230 | T/T | 285 (55.5%) | 379 (55.2%) | 1.00 | 0.98 |
|  | G/T | 198 (38.5%) | 268 (39%) | 0.98 (0.77-1.25) |
|  | G/G | 31 (6%) | 40 (5.8%) | 1.03 (0.63-1.69) |
|  | T | 768 (74.7%) | 1026 (74.7%) | 1.00 |  |
|  | G | 260 (25.3%) | 348 (25.3%) | 1.00 (0.83-1.20) | 0.98 |
| rs1159806 | A/A | 206 (40.1%) | 275 (40%) | 1.00 | 0.83 |
|  | A/T | 245 (47.7%) | 320 (46.6%) | 1.02 (0.80-1.31) |
|  | T/T | 63 (12.3%) | 92 (13.4%) | 0.91 (0.63-1.32) |
|  | A | 657 (63.9%) | 870 (63.3%) | 1.00 |  |
|  | T | 371 (36.1%) | 504 (36.7%) | 0.97 (0.82-1.15) | 0.76 |
| rs479744 | G/G | 357 (69.6%) | 479 (69.8%) | 1.00 | 0.63 |
|  | G/T | 144 (28.1%) | 185 (27%) | 1.04 (0.81-1.35) |
|  | T/T | 12 (2.3%) | 22 (3.2%) | 0.73 (0.36-1.50) |
|  | G | 858 (83.6%) | 1143 (83.3%) | 1.00 |  |
|  | T | 168 (16.4%) | 229 (16.7%) | 0.98 (0.79-1.21) | 0.84 |
| Significant results at *P*<0.05 are in bold. | | | | | |

| **Table C. Allele frequencies of the five male-longevity-associated *FOXO3* polymorphisms in the long-lived individuals and controls.** | | | | | | | | | | | |
| --- | --- | --- | --- | --- | --- | --- | --- | --- | --- | --- | --- |
| SNP No. | dbSNP ID | Allele | LLIs | | |  | Controls | | |  | *P*(male LLIs vs female controls) |
|  |  |  | Men | Women | *P* |  | Men | Women | *P* |  |  |
| SNP 4 | rs10499051 | A | 178 (87.3%) | 961 (93.5%) | **0.0037** |  | 295 (92.8%) | 1289 (93.8%) | 0.5 |  | **0.002** |
|  |  | G | 26 (12.7%) | 67 (6.5%) |  |  | 23 (7.2%) | 85 (6.2%) |  |  |  |
| SNP 10 | rs7762395 | G | 200 (98.0%) | 959 (93.3%) | **0.0041** |  | 299 (94.0%) | 1272 (92.6%) | 0.37 |  | **0.0011** |
|  |  | A | 4 (2.0%) | 69 (6.7%) |  |  | 19 (6.0%) | 102 (7.4%) |  |  |  |
| SNP 13 | rs4946933 | G | 178 (87.3%) | 960 (93.6%) | **0.0032** |  | 295 (92.8%) | 1282 (93.4%) | 0.67 |  | **0.0044** |
|  |  | A | 26 (12.7%) | 66 (6.4%) |  |  | 23 (7.2%) | 90 (6.6%) |  |  |  |
| SNP 14 | rs4945815 | C | 179 (87.7%) | 973 (94.8%) | **0.0004** |  | 300 (94.3%) | 1305 (95.0%) | 0.65 |  | **0.0003** |
|  |  | T | 25 (12.3%) | 53 (5.2%) |  |  | 18 (5.7%) | 69 (5.0%) |  |  |  |
| SNP 16 | rs3800230 | T | 132 (64.7%) | 768 (74.7%) | **0.0038** |  | 235 (73.9%) | 1026 (74.7%) | 0.77 |  | **0.003** |
|  |  | G | 72 (35.3%) | 260 (25.3%) |  |  | 83 (26.1%) | 348 (25.3%) |  |  |  |
| Significant results at *P*<0.05 are in bold. | | | | | | | | | | | |

| **Table D. Association of *FOXO3* haplotypes with human longevity.** | | | | | | | |
| --- | --- | --- | --- | --- | --- | --- | --- |
| Haplotype | rs768024-rs9486902-rs7746906-**rs10499051**-rs12206094-rs2802292-rs13220810-rs2764261-rs3813498-**rs7762395**-rs13207511-rs9400239-**rs4946933**-**rs4945815**-rs3800229-**rs3800230**-rs1159806-rs479744 |  | Frequency | | OR (95% CI) | *P* | *P*c |
|  |  |  | LLIs | Controls |  |  |  |
| All subjects | |  |  |  |  |  |  |
| 1 | G-C-G-**A-**C-T-T-G-T-**G-**A-C-**G-C-**T-**T-**A-G |  | 0.439 | 0.468 | 1.00 |  |  |
| 2 | A-C-A-**A-**T-G-T-A-T-**G-**A-T-**G-C-**G-***G-***T-G |  | 0.179 | 0.174 | 1.14 (0.92 - 1.42) | 0.22 | 1 |
| 3 | G-C-G-**A-**C-T-C-G-T-**G-**G-C-**G-C-**T-**T-**A-G |  | 0.0730 | 0.0731 | 1.16 (0.86 - 1.56) | 0.33 | 1 |
| 4 | G-T-G-**A-**T-G-T-A-C-***A-***A-T-**G-C-**G-**T-**T-T |  | 0.0470 | 0.0616 | 0.84 (0.60 - 1.18) | 0.31 | 1 |
| 5 | G-C-G-***G-***C-G-T-A-C-**G-**A-T-***A-T-***G-***G-***T-T |  | 0.0601 | 0.0484 | 1.37 (0.98 - 1.92) | 0.07 | 0.56 |
| 6 | G-C-G-**A-**C-T-T-G-T-**G-**G-C-**G-C-**T-**T-**A-G |  | 0.0556 | 0.0491 | 1.22 (0.87 - 1.71) | 0.24 | 1 |
| 7 | G-C-G-**A-**C-G-T-A-C-**G-**A-T-**G-C-**G-**T-**T-T |  | 0.0148 | 0.0160 | 1.13 (0.61 - 2.10) | 0.7 | 1 |
| 8 | G-C-G-**A-**C-T-T-G-T-**G-**A-C-**G-C-**T-**T-**T-T |  | 0.0137 | 0.0112 | 1.34 (0.67 - 2.65) | 0.41 | 1 |
| Rare haplotypes | |  | 0.112 | 0.0971 | 1.31 (0.97 - 1.76) | 0.082 | 0.656 |
| Global |  |  |  |  |  | 0.24 |  |
| Men | |  |  |  |  |  |  |
| 1 | G-C-G-**A-**C-T-T-G-T-**G-**A-C-**G-C-**T-**T-**A-G |  | 0.440 | 0.489 | 1.00 |  |  |
| 2 | A-C-A-**A-**T-G-T-A-T-**G-**A-T-**G-C-**G-***G-***T-G |  | 0.196 | 0.171 | 1.31 (0.81 - 2.12) | 0.28 | 1 |
| 3 | G-C-G-**A-**C-T-C-G-T-**G-**G-C-**G-C-**T-**T-**A-G |  | 0.0553 | 0.0902 | 0.76 (0.35 - 1.64) | 0.48 | 1 |
| 4 | G-T-G-**A-**T-G-T-A-C-***A-***A-T-**G-C-**G-**T-**T-T |  | 0.00980 | 0.0551 | 0.21 (0.05 - 0.95) | **0.043** | 0.258 |
| 5 | G-C-G-***G-***C-G-T-A-C-**G-**A-T-***A-T-***G-***G-***T-T |  | 0.114 | 0.0548 | 2.24 (1.09 - 4.58) | **0.028** | 0.168 |
| 6 | G-C-G-**A-**C-T-T-G-T-**G-**G-C-**G-C-**T-**T-**A-G |  | 0.0620 | 0.0401 | 1.50 (0.63 - 3.58) | 0.36 | 1 |
| Rare haplotypes | |  | 0.119 | 0.0994 | 1.49 (0.78 - 2.86) | 0.23 | 1 |
| Global |  |  |  |  |  | **0.0099** |  |
| Women | |  |  |  |  |  |  |
| 1 | G-C-G-**A-**C-T-T-G-T-**G-**A-C-**G-C-**T-**T-**A-G |  | 0.466 | 0.441 | 1.00 |  |  |
| 2 | A-C-A-**A-**T-G-T-A-T-**G-**A-T-**G-C-**G-***G-***T-G |  | 0.174 | 0.175 | 1.09 (0.86 - 1.39) | 0.46 | 1 |
| 3 | G-C-G-**A-**C-T-C-G-T-**G-**G-C-**G-C-**T-**T-**A-G |  | 0.0678 | 0.0747 | 1.24 (0.89 - 1.72) | 0.21 | 1 |
| 4 | G-T-G-**A-**T-G-T-A-C-***A-***A-T-**G-C-**G-**T-**T-T |  | 0.0629 | 0.0544 | 0.92 (0.65 - 1.32) | 0.67 | 1 |
| 5 | G-C-G-***G-***C-G-T-A-C-**G-**A-T-***A-T-***G-***G-***T-T |  | 0.0465 | 0.0490 | 1.15 (0.77 - 1.70) | 0.49 | 1 |
| 6 | G-C-G-**A-**C-T-T-G-T-**G-**G-C-**G-C-**T-**T-**A-G |  | 0.0501 | 0.0537 | 1.17 (0.80 - 1.69) | 0.42 | 1 |
| 7 | G-C-G-**A-**C-G-T-A-C-**G-**A-T-**G-C-**G-**T-**T-T |  | 0.0159 | 0.0157 | 1.07 (0.54 - 2.12) | 0.84 | 1 |
| 8 | G-C-G-**A-**C-T-T-G-T-**G-**A-C-**G-C-**T-**T-**T-T |  | 0.0114 | 0.0144 | 1.34 (0.64 - 2.81) | 0.45 | 1 |
| Rare haplotypes | |  | 0.103 | 0.120 | 1.25 (0.90 - 1.74) | 0.18 | 1 |
| Global |  |  |  |  |  | 0.78 |  |
| Significant results at *P*<0.05 are in bold. For the five SNPs (rs10499051, rs7762395, rs4946933, rs4945815 and rs3800230) significantly associated with male longevity, alleles are in bold and alleles different from the corresponding alleles in Haplotype 1 (G-C-G-**A-**C-T-T-G-T-**G-**A-C-**G-C-**T-**T-**A-G) are in bold and italic. *P*c, *P* values after Bonferroni correction using the number of haplotypes tested (n = 8, 6, 8 for all subjects, men and women, respectively). | | | | | | | |

| **Table E. Haplotype frequencies of male-longevity-associated SNPs in *FOXO3* in the long-lived individuals and controls.** | | | | | | | | | | | |
| --- | --- | --- | --- | --- | --- | --- | --- | --- | --- | --- | --- |
| Haplotype | rs10499051-rs7762395-rs4945815-rs3800230 |  | LLIs | | |  | Controls | | |  | *P* (male LLIs vs female controls) |
|  |  |  | Frequency | | *P* |  | Frequency | | *P* |  |  |
|  |  |  | Men | Women |  |  | Men | Women |  |  |  |
| 1 | A-G-C-T |  | 0.628 | 0.670 |  |  | 0.673 | 0.662 |  |  |  |
| 2 | A-G-C-***G*** |  | 0.226 | 0.198 | 0.19 |  | 0.197 | 0.201 | 0.86 |  | 0.35 |
| 3 | A-***A***-C-T |  | 0.0196 | 0.0664 | 0.041 |  | 0.0570 | 0.0737 | 0.33 |  | 0.02 |
| 4 | ***G***-G-***T***-***G*** |  | 0.123 | 0.0516 | **0.0004** |  | 0.0566 | 0.0480 | 0.64 |  | **0.0002** |
| 5 | ***G***-G-C-T |  |  |  |  |  | 0.00860 | 0.0113 | 0.77 |  | 1 |
| Rare haplotypes | |  | 0.00490 | 0.0136 | 0.36 |  | 0.00720 | 0.00480 | 0.84 |  | 0.86 |
| Global |  |  |  |  | **0.00022** |  |  |  | 0.92 |  | **<0.0001** |
| Significant results at *P*<0.05 are in bold. Alleles in bold and italic are different from the corresponding alleles in Haplotype 1 (A-G-C-T). | | | | | | | | | | | |

| **Table F. Genotype and allele frequencies of *FOXO3* polymorphisms in the Chinese Han long-lived individuals and controls.** | | | | | |
| --- | --- | --- | --- | --- | --- |
| dbSNP ID | Genotype/Allele | LLIs | Controls | OR(95%CI) | *P* |
| rs768024 | G/G | 373 (64.5%) | 503 (64.7%) | 1.00 | 0.1 |
|  | G/A | 177 (30.6%) | 253 (32.6%) | 0.94 (0.75-1.19) |
|  | A/A | 28 (4.8%) | 21 (2.7%) | **1.80 (1.01-3.22)** |
|  | G | 923 (79.8%) | 1259 (81.0%) | 1.00 |  |
|  | A | 233 (20.2%) | 295 (19.0%) | 1.08 (0.89-1.31) | 0.44 |
| rs9486902 | C/C | 491 (85.5%) | 657 (85.3%) | 1.00 | 0.43 |
|  | C/T | 81 (14.1%) | 106 (13.8%) | 1.02 (0.75-1.40) |
|  | T/T | 2 (0.4%) | 7 (0.9%) | 0.38 (0.08-1.85) |
|  | C | 1063 (92.6%) | 1420 (92.2%) | 1.00 |  |
|  | T | 85 (7.4%) | 120 (7.8%) | 0.95 (0.71-1.26) | 0.71 |
| rs7746906 | G/G | 361 (62.9%) | 502 (64.6%) | 1.00 | 0.11 |
|  | G/A | 185 (32.2%) | 254 (32.7%) | 1.01 (0.80-1.28) |
|  | A/A | 28 (4.9%) | 21 (2.7%) | **1.85 (1.04-3.32)** |
|  | G | 907 (79.0%) | 1258 (81.0%) | 1.00 |  |
|  | A | 241 (21.0%) | 296 (19.0%) | 1.13 (0.93-1.37) | 0.21 |
| rs10499051 | A/A | 496 (85.8%) | 679 (87.4%) | 1.00 | 0.63 |
|  | G/A | 79 (13.7%) | 93 (12%) | 1.16 (0.84-1.60) |
|  | G/G | 3 (0.5%) | 5 (0.6%) | 0.82 (0.20-3.45) |
|  | A | 1071 (92.6%) | 1451 (93.4%) | 1.00 |  |
|  | G | 85 (7.4%) | 103 (6.6%) | 1.12 (0.83-1.50) | 0.47 |
| rs12206094 | C/C | 328 (56.9%) | 424 (54.6%) | 1.00 | 0.29 |
|  | C/T | 201 (34.8%) | 299 (38.5%) | 0.87 (0.69-1.09) |
|  | T/T | 48 (8.3%) | 53 (6.8%) | 1.17 (0.77-1.78) |
|  | C | 857 (74.3%) | 1147 (73.9%) | 1.00 |  |
|  | T | 297 (25.7%) | 405 (26.1%) | 0.98 (0.83-1.16) | 0.84 |
| rs2802292 | T/T | 237 (41.5%) | 324 (42.1%) | 1.00 | 0.95 |
|  | G/T | 268 (46.9%) | 355 (46.1%) | 1.03 (0.82-1.30) |
|  | G/G | 66 (11.6%) | 91 (11.8%) | 0.99 (0.69-1.42) |
|  | T | 742 (65.0%) | 1003 (65.1%) | 1.00 |  |
|  | G | 400 (35.0%) | 537 (34.9%) | 1.01 (0.86-1.18) | 0.93 |
| rs13220810 | T/T | 477 (82.5%) | 654 (84.3%) | 1.00 | 0.32 |
|  | C/T | 94 (16.3%) | 118 (15.2%) | 1.09 (0.81-1.47) |
|  | C/C | 7 (1.2%) | 4 (0.5%) | 2.40 (0.70-8.24) |
|  | T | 1048 (90.7%) | 1426 (91.9%) | 1.00 |  |
|  | C | 108 (9.3%) | 126 (8.1%) | 1.16 (0.89-1.52) | 0.27 |
| rs2764261 | G/G | 245 (42.4%) | 327 (42.1%) | 1.00 | 0.96 |
|  | G/A | 269 (46.5%) | 360 (46.3%) | 1.00 (0.79-1.25) |
|  | A/A | 64 (11.1%) | 90 (11.6%) | 0.95 (0.66-1.36) |
|  | G | 759 (65.7%) | 1014 (65.3%) | 1.00 |  |
|  | A | 397 (34.3%) | 540 (34.7%) | 0.98 (0.83-1.15) | 0.82 |
| rs3813498 | T/T | 379 (65.9%) | 531 (68.5%) | 1.00 | 0.26 |
|  | C/T | 182 (31.6%) | 218 (28.1%) | 1.17 (0.92-1.48) |
|  | C/C | 14 (2.4%) | 26 (3.4%) | 0.75 (0.39-1.46) |
|  | T | 940 (81.7%) | 1280 (82.6%) | 1.00 |  |
|  | C | 210 (18.3%) | 270 (17.4%) | 1.06 (0.87-1.30) | 0.57 |
| rs7762395 | G/G | 517 (89.5%) | 675 (86.9%) | 1.00 | 0.35 |
|  | G/A | 57 (9.9%) | 95 (12.2%) | 0.78 (0.55-1.11) |
|  | A/A | 4 (0.7%) | 7 (0.9%) | 0.75 (0.22-2.56) |
|  | G | 1091 (94.4%) | 1445 (93.0%) | 1.00 |  |
|  | A | 65 (5.6%) | 109 (7.0%) | 0.80 (0.59-1.09) | 0.15 |
| rs13207511 | A/A | 410 (70.9%) | 580 (74.7%) | 1.00 | 0.31 |
|  | G/A | 156 (27%) | 184 (23.7%) | 1.20 (0.94-1.54) |
|  | G/G | 12 (2.1%) | 13 (1.7%) | 1.31 (0.59-2.89) |
|  | A | 976 (84.4%) | 1344 (86.5%) | 1.00 |  |
|  | G | 180 (15.6%) | 210 (13.5%) | 1.18 (0.95-1.47) | 0.13 |
| rs9400239 | C/C | 244 (42.2%) | 318 (40.9%) | 1.00 | 0.85 |
|  | C/T | 268 (46.4%) | 364 (46.9%) | 0.96 (0.76-1.21) |
|  | T/T | 66 (11.4%) | 95 (12.2%) | 0.91 (0.63-1.29) |
|  | C | 756 (65.4%) | 1000 (64.4%) | 1.00 |  |
|  | T | 400 (34.6%) | 554 (35.6%) | 0.95 (0.81-1.12) | 0.57 |
| rs4946933 | G/G | 496 (86%) | 674 (86.9%) | 1.00 | 0.71 |
|  | G/A | 78 (13.5%) | 96 (12.4%) | 1.10 (0.80-1.52) |
|  | A/A | 3 (0.5%) | 6 (0.8%) | 0.68 (0.17-2.73) |
|  | G | 1070 (92.7%) | 1444 (93.0%) | 1.00 |  |
|  | A | 84 (7.3%) | 108 (7.0%) | 1.05 (0.78-1.40) | 0.75 |
| rs4945815 | C/C | 508 (88%) | 699 (90%) | 1.00 | 0.42 |
|  | C/T | 67 (11.6%) | 74 (9.5%) | 1.25 (0.88-1.77) |
|  | T/T | 2 (0.4%) | 4 (0.5%) | 0.69 (0.13-3.77) |
|  | C | 1083 (93.8%) | 1472 (94.7%) | 1.00 |  |
|  | T | 71 (6.2%) | 82 (5.3%) | 1.17 (0.85-1.62) | 0.34 |
| rs3800229 | T/T | 246 (42.7%) | 323 (41.8%) | 1.00 | 0.86 |
|  | G/T | 266 (46.2%) | 357 (46.2%) | 0.98 (0.78-1.23) |
|  | G/G | 64 (11.1%) | 93 (12%) | 0.90 (0.63-1.29) |
|  | T | 758 (65.8%) | 1003 (64.9%) | 1.00 |  |
|  | G | 394 (34.2%) | 543 (35.1%) | 0.96 (0.82-1.13) | 0.62 |
| rs3800230 | T/T | 311 (53.8%) | 425 (54.7%) | 1.00 | 0.86 |
|  | G/T | 228 (39.5%) | 305 (39.2%) | 1.02 (0.82-1.28) |
|  | G/G | 39 (6.8%) | 47 (6%) | 1.13 (0.72-1.78) |
|  | T | 850 (73.5%) | 1155 (74.3%) | 1.00 |  |
|  | G | 306 (26.5%) | 399 (25.7%) | 1.04 (0.88-1.24) | 0.64 |
| rs1159806 | A/A | 232 (40.1%) | 312 (40.1%) | 1.00 | 0.94 |
|  | A/T | 273 (47.2%) | 362 (46.6%) | 1.01 (0.80-1.28) |
|  | T/T | 73 (12.6%) | 103 (13.3%) | 0.95 (0.68-1.35) |
|  | A | 737 (63.8%) | 986 (63.4%) | 1.00 |  |
|  | T | 419 (36.2%) | 568 (36.6%) | 0.99 (0.84-1.16) | 0.87 |
| rs479744 | G/G | 402 (69.7%) | 541 (69.7%) | 1.00 | 0.49 |
|  | G/T | 163 (28.2%) | 211 (27.2%) | 1.04 (0.82-1.32) |
|  | T/T | 12 (2.1%) | 24 (3.1%) | 0.67 (0.33-1.36) |
|  | G | 967 (83.8%) | 1293 (83.3%) | 1.00 |  |
|  | T | 187 (16.2%) | 259 (16.7%) | 0.97 (0.79-1.19) | 0.74 |

| **Table G. Genotype and allele frequencies of *FOXO3* polymorphisms in the Chinese Han long-lived individuals and controls when stratifying by gender.** | | | | | |
| --- | --- | --- | --- | --- | --- |
| dbSNP ID | Genotype/Allele | LLIs | Controls | OR(95%CI) | *P* |
| Men |  |  |  |  |  |
| rs768024 | G/G | 62 (64.6%) | 96 (65.3%) | 1.00 | 0.38 |
|  | G/A | 27 (28.1%) | 46 (31.3%) | 0.91 (0.51-1.61) |
|  | A/A | 7 (7.3%) | 5 (3.4%) | 2.17 (0.66-7.13) |
|  | G | 151 (78.6%) | 238 (81.0%) | 1.00 |  |
|  | A | 41 (21.4%) | 56 (19.0%) | 1.14 (0.74-1.78) | 0.55 |
| rs9486902 | C/C | 87 (90.6%) | 130 (88.4%) | 1.00 | 0.56 |
|  | C/T | 9 (9.4%) | 16 (10.9%) | 0.84 (0.36-1.99) |
|  | T/T | 0 (0%) | 1 (0.7%) |  |
|  | C | 183 (95.3%) | 276 (93.9%) | 1.00 |  |
|  | T | 9 (4.7%) | 18 (6.1%) | 0.76 (0.34-1.71) | 0.5 |
| rs7746906 | G/G | 58 (61.7%) | 97 (66%) | 1.00 | 0.37 |
|  | G/A | 29 (30.9%) | 45 (30.6%) | 1.08 (0.61-1.90) |
|  | A/A | 7 (7.5%) | 5 (3.4%) | 2.34 (0.71-7.72) |
|  | G | 145 (77.1%) | 239 (81.3%) | 1.00 |  |
|  | A | 43 (22.9%) | 55 (18.7%) | 1.27 (0.82-1.98) | 0.28 |
| rs10499051 | A/A | 72 (75%) | 125 (85%) | 1.00 | 0.08 |
|  | G/A | 23 (24%) | 22 (15%) | 1.82 (0.95-3.49) |
|  | G/G | 1 (1%) | 0 (0%) |  |
|  | A | 167 (87.0%) | 272 (92.5%) | 1.00 |  |
|  | G | 25 (13.0%) | 22 (7.5%) | **1.93 (1.03-3.63)** | **0.039** |
| rs12206094 | C/C | 58 (60.4%) | 84 (57.1%) | 1.00 | 0.71 |
|  | C/T | 30 (31.2%) | 53 (36%) | 0.82 (0.47-1.43) |
|  | T/T | 8 (8.3%) | 10 (6.8%) | 1.16 (0.43-3.11) |
|  | C | 146 (76.0%) | 221 (75.2%) | 1.00 |  |
|  | T | 46 (24.0%) | 73 (24.8%) | 0.96 (0.64-1.44) | 0.83 |
| rs2802292 | T/T | 36 (37.5%) | 62 (42.2%) | 1.00 | 0.54 |
|  | G/T | 46 (47.9%) | 70 (47.6%) | 1.13 (0.65-1.97) |
|  | G/G | 14 (14.6%) | 15 (10.2%) | 1.61 (0.70-3.71) |
|  | T | 118 (61.5%) | 194 (66.0%) | 1.00 |  |
|  | G | 74 (38.5%) | 100 (34.0%) | 1.23 (0.83-1.81) | 0.3 |
| rs13220810 | T/T | 80 (83.3%) | 119 (81%) | 1.00 | 0.57 |
|  | C/T | 16 (16.7%) | 27 (18.4%) | 0.88 (0.45-1.74) |
|  | C/C | 0 (0%) | 1 (0.7%) |  |
|  | T | 176 (91.7%) | 265 (90.1%) | 1.00 |  |
|  | C | 16 (8.3%) | 29 (9.9%) | 0.82 (0.43-1.59) | 0.56 |
| rs2764261 | G/G | 36 (37.5%) | 62 (42.2%) | 1.00 | 0.7 |
|  | G/A | 47 (49%) | 69 (46.9%) | 1.17 (0.67-2.04) |
|  | A/A | 13 (13.5%) | 16 (10.9%) | 1.40 (0.60-3.24) |
|  | G | 119 (62.0%) | 193 (65.6%) | 1.00 |  |
|  | A | 73 (38.0%) | 101 (34.4%) | 1.18 (0.80-1.74) | 0.4 |
| rs3813498 | T/T | 61 (63.5%) | 105 (71.4%) | 1.00 | 0.42 |
|  | C/T | 33 (34.4%) | 39 (26.5%) | 1.46 (0.83-2.55) |
|  | C/C | 2 (2.1%) | 3 (2%) | 1.15 (0.19-7.06) |
|  | T | 155 (80.7%) | 249 (84.7%) | 1.00 |  |
|  | C | 37 (19.3%) | 45 (15.3%) | 1.34 (0.82-2.21) | 0.24 |
| rs7762395 | G/G | 92 (95.8%) | 131 (89.1%) | 1.00 | 0.12 |
|  | G/A | 4 (4.2%) | 15 (10.2%) | 0.38 (0.12-1.18) |
|  | A/A | 0 (0%) | 1 (0.7%) |  |
|  | G | 188 (97.9%) | 277 (94.2%) | 1.00 |  |
|  | A | 4 (2.1%) | 17 (5.8%) | 0.36 (0.12-1.08) | **0.044** |
| rs13207511 | A/A | 67 (69.8%) | 104 (70.8%) | 1.00 | 0.95 |
|  | G/A | 28 (29.2%) | 42 (28.6%) | 1.03 (0.59-1.83) |
|  | G/G | 1 (1%) | 1 (0.7%) | 1.55 (0.10-25.24) |
|  | A | 162 (84.4%) | 250 (85.0%) | 1.00 |  |
|  | G | 30 (15.6%) | 44 (15.0%) | 1.06 (0.62-1.81) | 0.83 |
| rs9400239 | C/C | 37 (38.5%) | 60 (40.8%) | 1.00 | 0.78 |
|  | C/T | 45 (46.9%) | 70 (47.6%) | 1.04 (0.60-1.82) |
|  | T/T | 14 (14.6%) | 17 (11.6%) | 1.34 (0.59-3.02) |
|  | C | 119 (62.0%) | 190 (64.6%) | 1.00 |  |
|  | T | 73 (38.0%) | 104 (35.4%) | 1.12 (0.77-1.65) | 0.55 |
| rs4946933 | G/G | 72 (75%) | 125 (85%) | 1.00 | 0.08 |
|  | G/A | 23 (24%) | 22 (15%) | 1.82 (0.95-3.49) |
|  | A/A | 1 (1%) | 0 (0%) |  |
|  | G | 167 (87.0%) | 272 (92.5%) | 1.00 |  |
|  | A | 25 (13.0%) | 22 (7.5%) | **1.93 (1.03-3.63)** | **0.039** |
| rs4945815 | C/C | 73 (76%) | 130 (88.4%) | 1.00 | **0.024** |
|  | C/T | 22 (22.9%) | 17 (11.6%) | **2.30 (1.15-4.62)** |
|  | T/T | 1 (1%) | 0 (0%) |  |
|  | C | 168 (87.5%) | 277 (94.2%) | 1.00 |  |
|  | T | 24 (12.5%) | 17 (5.8%) | **2.43 (1.24-4.76)** | **0.0085** |
| rs3800229 | T/T | 37 (38.5%) | 60 (40.8%) | 1.00 | 0.56 |
|  | G/T | 44 (45.8%) | 71 (48.3%) | 1.00 (0.58-1.75) |
|  | G/G | 15 (15.6%) | 16 (10.9%) | 1.52 (0.67-3.43) |
|  | T | 118 (61.5%) | 191 (65.0%) | 1.00 |  |
|  | G | 74 (38.5%) | 103 (35.0%) | 1.17 (0.80-1.71) | 0.43 |
| rs3800230 | T/T | 42 (43.8%) | 78 (53.1%) | 1.00 | 0.2 |
|  | G/T | 43 (44.8%) | 60 (40.8%) | 1.33 (0.77-2.29) |
|  | G/G | 11 (11.5%) | 9 (6.1%) | 2.27 (0.87-5.91) |
|  | T | 127 (66.1%) | 216 (73.5%) | 1.00 |  |
|  | G | 65 (33.9%) | 78 (26.5%) | 1.43 (0.96-2.14) | 0.081 |
| rs1159806 | A/A | 36 (37.5%) | 60 (40.8%) | 1.00 | 0.32 |
|  | A/T | 43 (44.8%) | 71 (48.3%) | 1.01 (0.58-1.77) |
|  | T/T | 17 (17.7%) | 16 (10.9%) | 1.77 (0.80-3.93) |
|  | A | 115 (59.9%) | 191 (65.0%) | 1.00 |  |
|  | T | 77 (40.1%) | 103 (35.0%) | 1.24 (0.85-1.81) | 0.26 |
| rs479744 | G/G | 64 (66.7%) | 104 (70.8%) | 1.00 | 0.76 |
|  | G/T | 30 (31.2%) | 41 (27.9%) | 1.19 (0.68-2.09) |
|  | T/T | 2 (2.1%) | 2 (1.4%) | 1.62 (0.22-11.82) |
|  | G | 158 (82.3%) | 249 (84.7%) | 1.00 |  |
|  | T | 34 (17.7%) | 45 (15.3%) | 1.21 (0.73-2.01) | 0.47 |
| Women |  |  |  |  |  |
| rs768024 | G/G | 311 (64.5%) | 407 (64.6%) | 1.00 | 0.23 |
|  | G/A | 150 (31.1%) | 207 (32.9%) | 0.95 (0.73-1.23) |
|  | A/A | 21 (4.4%) | 16 (2.5%) | 1.72 (0.88-3.35) |
|  | G | 772 (80.1%) | 1021 (81.0%) | 1.00 |  |
|  | A | 192 (19.9%) | 239 (19.0%) | 1.06 (0.86-1.32) | 0.57 |
| rs9486902 | C/C | 404 (84.5%) | 527 (84.6%) | 1.00 | 0.54 |
|  | C/T | 72 (15.1%) | 90 (14.4%) | 1.04 (0.75-1.46) |
|  | T/T | 2 (0.4%) | 6 (1%) | 0.43 (0.09-2.17) |
|  | C | 880 (92.1%) | 1144 (91.8%) | 1.00 |  |
|  | T | 76 (7.9%) | 102 (8.2%) | 0.97 (0.71-1.32) | 0.84 |
| rs7746906 | G/G | 303 (63.1%) | 405 (64.3%) | 1.00 | 0.24 |
|  | G/A | 156 (32.5%) | 209 (33.2%) | 1.00 (0.77-1.29) |
|  | A/A | 21 (4.4%) | 16 (2.5%) | 1.75 (0.90-3.42) |
|  | G | 762 (79.4%) | 1019 (80.9%) | 1.00 |  |
|  | A | 198 (20.6%) | 241 (19.1%) | 1.10 (0.89-1.37) | 0.37 |
| rs10499051 | A/A | 424 (88%) | 554 (87.9%) | 1.00 | 0.71 |
|  | G/A | 56 (11.6%) | 71 (11.3%) | 1.03 (0.71-1.50) |
|  | G/G | 2 (0.4%) | 5 (0.8%) | 0.52 (0.10-2.71) |
|  | A | 904 (93.8%) | 1179 (93.6%) | 1.00 |  |
|  | G | 60 (6.2%) | 81 (6.4%) | 0.97 (0.69-1.36) | 0.85 |
| rs12206094 | C/C | 270 (56.1%) | 340 (54%) | 1.00 | 0.38 |
|  | C/T | 171 (35.5%) | 246 (39.1%) | 0.88 (0.68-1.13) |
|  | T/T | 40 (8.3%) | 43 (6.8%) | 1.17 (0.74-1.85) |
|  | C | 711 (73.9%) | 926 (73.6%) | 1.00 |  |
|  | T | 251 (26.1%) | 332 (26.4%) | 0.99 (0.82-1.19) | 0.88 |
| rs2802292 | T/T | 201 (42.3%) | 262 (42%) | 1.00 | 0.81 |
|  | G/T | 222 (46.7%) | 285 (45.8%) | 1.02 (0.79-1.31) |
|  | G/G | 52 (10.9%) | 76 (12.2%) | 0.89 (0.60-1.33) |
|  | T | 624 (65.7%) | 809 (64.9%) | 1.00 |  |
|  | G | 326 (34.3%) | 437 (35.1%) | 0.97 (0.81-1.16) | 0.71 |
| rs13220810 | T/T | 397 (82.4%) | 535 (85.1%) | 1.00 | 0.16 |
|  | C/T | 78 (16.2%) | 91 (14.5%) | 1.16 (0.83-1.61) |
|  | C/C | 7 (1.4%) | 3 (0.5%) | 3.14 (0.81-12.24) |
|  | T | 872 (90.5%) | 1161 (92.3%) | 1.00 |  |
|  | C | 92 (9.5%) | 97 (7.7%) | 1.26 (0.93-1.69) | 0.13 |
| rs2764261 | G/G | 209 (43.4%) | 265 (42.1%) | 1.00 | 0.8 |
|  | G/A | 222 (46.1%) | 291 (46.2%) | 0.97 (0.75-1.24) |
|  | A/A | 51 (10.6%) | 74 (11.8%) | 0.87 (0.59-1.30) |
|  | G | 640 (66.4%) | 821 (65.2%) | 1.00 |  |
|  | A | 324 (33.6%) | 439 (34.8%) | 0.95 (0.79-1.13) | 0.54 |
| rs3813498 | T/T | 318 (66.4%) | 426 (67.8%) | 1.00 | 0.39 |
|  | C/T | 149 (31.1%) | 179 (28.5%) | 1.12 (0.86-1.45) |
|  | C/C | 12 (2.5%) | 23 (3.7%) | 0.70 (0.34-1.43) |
|  | T | 785 (81.9%) | 1031 (82.1%) | 1.00 |  |
|  | C | 173 (18.1%) | 225 (17.9%) | 1.01 (0.81-1.26) | 0.93 |
| rs7762395 | G/G | 425 (88.2%) | 544 (86.3%) | 1.00 | 0.66 |
|  | G/A | 53 (11%) | 80 (12.7%) | 0.85 (0.59-1.23) |
|  | A/A | 4 (0.8%) | 6 (1%) | 0.85 (0.24-3.04) |
|  | G | 903 (93.7%) | 1168 (92.7%) | 1.00 |  |
|  | A | 61 (6.3%) | 92 (7.3%) | 0.87 (0.63-1.20) | 0.38 |
| rs13207511 | A/A | 343 (71.2%) | 476 (75.6%) | 1.00 | 0.26 |
|  | G/A | 128 (26.6%) | 142 (22.5%) | 1.25 (0.95-1.65) |
|  | G/G | 11 (2.3%) | 12 (1.9%) | 1.27 (0.55-2.92) |
|  | A | 814 (84.4%) | 1094 (86.8%) | 1.00 |  |
|  | G | 150 (15.6%) | 166 (13.2%) | 1.21 (0.96-1.54) | 0.11 |
| rs9400239 | C/C | 207 (43%) | 258 (41%) | 1.00 | 0.65 |
|  | C/T | 223 (46.3%) | 294 (46.7%) | 0.95 (0.73-1.22) |
|  | T/T | 52 (10.8%) | 78 (12.4%) | 0.83 (0.56-1.23) |
|  | C | 637 (66.1%) | 810 (64.3%) | 1.00 |  |
|  | T | 327 (33.9%) | 450 (35.7%) | 0.92 (0.77-1.10) | 0.37 |
| rs4946933 | G/G | 424 (88.2%) | 549 (87.3%) | 1.00 | 0.55 |
|  | G/A | 55 (11.4%) | 74 (11.8%) | 0.96 (0.66-1.39) |
|  | A/A | 2 (0.4%) | 6 (1%) | 0.43 (0.09-2.15) |
|  | G | 903 (93.9%) | 1172 (93.2%) | 1.00 |  |
|  | A | 59 (6.1%) | 86 (6.8%) | 0.90 (0.64-1.25) | 0.52 |
| rs4945815 | C/C | 435 (90.4%) | 569 (90.3%) | 1.00 | 0.54 |
|  | C/T | 45 (9.4%) | 57 (9.1%) | 1.03 (0.69-1.56) |
|  | T/T | 1 (0.2%) | 4 (0.6%) | 0.33 (0.04-2.94) |
|  | C | 915 (95.1%) | 1195 (94.8%) | 1.00 |  |
|  | T | 47 (4.9%) | 65 (5.2%) | 0.95 (0.65-1.38) | 0.77 |
| rs3800229 | T/T | 209 (43.5%) | 263 (42%) | 1.00 | 0.54 |
|  | G/T | 222 (46.2%) | 286 (45.7%) | 0.98 (0.76-1.26) |
|  | G/G | 49 (10.2%) | 77 (12.3%) | 0.80 (0.54-1.20) |
|  | T | 640 (66.7%) | 812 (64.9%) | 1.00 |  |
|  | G | 320 (33.3%) | 440 (35.1%) | 0.92 (0.77-1.10) | 0.37 |
| rs3800230 | T/T | 269 (55.8%) | 347 (55.1%) | 1.00 | 0.97 |
|  | G/T | 185 (38.4%) | 245 (38.9%) | 0.97 (0.76-1.25) |
|  | G/G | 28 (5.8%) | 38 (6%) | 0.95 (0.57-1.59) |
|  | T | 723 (75.0%) | 939 (74.5%) | 1.00 |  |
|  | G | 241 (25.0%) | 321 (25.5%) | 0.97 (0.80-1.18) | 0.8 |
| rs1159806 | A/A | 196 (40.7%) | 252 (40%) | 1.00 | 0.55 |
|  | A/T | 230 (47.7%) | 291 (46.2%) | 1.02 (0.79-1.31) |
|  | T/T | 56 (11.6%) | 87 (13.8%) | 0.83 (0.56-1.22) |
|  | A | 622 (64.5%) | 795 (63.1%) | 1.00 |  |
|  | T | 342 (35.5%) | 465 (36.9%) | 0.94 (0.79-1.12) | 0.48 |
| rs479744 | G/G | 338 (70.3%) | 437 (69.5%) | 1.00 | 0.36 |
|  | G/T | 133 (27.6%) | 170 (27%) | 1.01 (0.77-1.32) |
|  | T/T | 10 (2.1%) | 22 (3.5%) | 0.59 (0.27-1.26) |
|  | G | 809 (84.1%) | 1044 (83.0%) | 1.00 |  |
|  | T | 153 (15.9%) | 214 (17.0%) | 0.92 (0.74-1.16) | 0.49 |
| Significant results at *P*<0.05 are in bold. | | | | | |

| **Table H. Allele frequencies of the five male-longevity-associated *FOXO3* polymorphisms in the Chinese Han long-lived individuals and controls.** | | | | | | | | | | | |
| --- | --- | --- | --- | --- | --- | --- | --- | --- | --- | --- | --- |
| SNP No. | dbSNP ID | Allele | LLIs | | |  | Controls | | |  | *P*(male LLIs vs female controls) |
|  |  |  | Men | Women | *P* |  | Men | Women | *P* |  |  |
| SNP 4 | rs10499051 | A | 167 (87.0%) | 904 (93.8%) | **0.0021** |  | 272 (92.5%) | 1179 (93.6%) | 0.53 |  | **0.0031** |
|  |  | G | 25 (13.0%) | 60 (6.2%) |  |  | 22 (7.5%) | 81 (6.4%) |  |  |  |
| SNP 10 | rs7762395 | G | 188 (97.9%) | 903 (93.7%) | **0.011** |  | 277 (94.2%) | 1168 (92.7%) | 0.36 |  | **0.0026** |
|  |  | A | 4 (2.1%) | 61 (6.3%) |  |  | 17 (5.8%) | 92 (7.3%) |  |  |  |
| SNP 13 | rs4946933 | G | 167 (87.0%) | 903 (93.9%) | **0.0018** |  | 272 (92.5%) | 1172 (93.2%) | 0.7 |  | **0.0066** |
|  |  | A | 25 (13.0%) | 59 (6.1%) |  |  | 22 (7.5%) | 86 (6.8%) |  |  |  |
| SNP 14 | rs4945815 | C | 168 (87.5%) | 915 (95.1%) | **0.0002** |  | 277 (94.2%) | 1195 (94.8%) | 0.68 |  | **0.0005** |
|  |  | T | 24 (12.5%) | 47 (4.9%) |  |  | 17 (5.8%) | 65 (5.2%) |  |  |  |
| SNP 16 | rs3800230 | T | 127 (66.1%) | 723 (75.0%) | **0.012** |  | 216 (73.5%) | 939 (74.5%) | 0.71 |  | **0.016** |
|  |  | G | 65 (33.9%) | 241 (25.0%) |  |  | 78 (26.5%) | 321 (25.5%) |  |  |  |
| Significant results at *P*<0.05 are in bold. | | | | | | | | | | | |

| **Table I. Association of *FOXO3* haplotypes with human longevity in the Chinese Han population.** | | | | | | | |
| --- | --- | --- | --- | --- | --- | --- | --- |
| Haplotype | rs768024-rs9486902-rs7746906-**rs10499051**-rs12206094-rs2802292-rs13220810-rs2764261-rs3813498-**rs7762395**-rs13207511-rs9400239-**rs4946933**-**rs4945815**-rs3800229-**rs3800230**-rs1159806-rs479744 |  | Frequency | | OR (95% CI) | *P* | *P*c |
|  |  |  | LLIs | Controls |  |  |  |
| All subjects | |  |  |  |  |  |  |
| 1 | G-C-G-**A-**C-T-T-G-T-**G-**A-C-**G-C-**T-**T-**A-G |  | 0.443 | 0.465 | 1.00 |  |  |
| 2 | A-C-A-**A-**T-G-T-A-T-**G-**A-T-**G-C-**G-***G-***T-G |  | 0.175 | 0.173 | 1.11 (0.89 - 1.38) | 0.37 | 1 |
| 3 | G-C-G-**A-**C-T-C-G-T-**G-**G-C-**G-C-**T-**T-**A-G |  | 0.0730 | 0.0727 | 1.14 (0.83 - 1.56) | 0.41 | 1 |
| 4 | G-T-G-**A-**T-G-T-A-C-***A-***A-T-**G-C-**G-**T-**T-T |  | 0.0440 | 0.0608 | 0.79 (0.55 - 1.13) | 0.19 | 1 |
| 5 | G-C-G-***G-***C-G-T-A-C-**G-**A-T-***A-T-***G-***G-***T-T |  | 0.0589 | 0.0495 | 1.27 (0.90 - 1.81) | 0.17 | 1 |
| 6 | G-C-G-**A-**C-T-T-G-T-**G-**G-C-**G-C-**T-**T-**A-G |  | 0.0592 | 0.0502 | 1.25 (0.89 - 1.77) | 0.19 | 1 |
| 7 | G-C-G-**A-**C-G-T-A-C-**G-**A-T-**G-C-**G-**T-**T-T |  | 0.0147 | 0.0152 | 1.16 (0.60 - 2.25) | 0.65 | 1 |
| 8 | G-C-G-**A-**C-T-T-G-T-**G-**A-C-**G-C-**T-**T-**T-T |  | 0.0144 | 0.0111 | 1.36 (0.67 - 2.75) | 0.4 | 1 |
| Rare haplotypes | |  | 0.114 | 0.100 | 1.23 (0.90 - 1.68) | 0.2 | 1 |
| Global |  |  |  |  |  | 0.38 |  |
| Men | |  |  |  |  |  |  |
| 1 | G-C-G-**A-**C-T-T-G-T-**G-**A-C-**G-C-**T-**T-**A-G |  | 0.441 | 0.502 | 1.00 |  |  |
| 2 | A-C-A-**A-**T-G-T-A-T-**G-**A-T-**G-C-**G-***G-***T-G |  | 0.182 | 0.169 | 1.22 (0.74 - 2.03) | 0.44 | 1 |
| 3 | G-C-G-**A-**C-T-C-G-T-**G-**G-C-**G-C-**T-**T-**A-G |  | 0.0589 | 0.0915 | 0.78 (0.36 - 1.69) | 0.53 | 1 |
| 4 | G-T-G-**A-**T-G-T-A-C-***A-***A-T-**G-C-**G-**T-**T-T |  | 0.0104 | 0.0527 | 0.24 (0.05 - 1.09) | 0.066 | 0.396 |
| 5 | G-C-G-***G-***C-G-T-A-C-**G-**A-T-***A-T-***G-***G-***T-T |  | 0.116 | 0.0545 | **2.27 (1.08 - 4.79)** | **0.032** | 0.192 |
| 6 | G-C-G-**A-**C-T-T-G-T-**G-**G-C-**G-C-**T-**T-**A-G |  | 0.0661 | 0.0321 | 1.79 (0.71 - 4.53) | 0.22 | 1 |
| Rare haplotypes | |  | 0.121 | 0.0961 | 1.67 (0.86 - 3.26) | 0.13 | 0.78 |
| Global |  |  |  |  |  | **0.015** |  |
| Women | |  |  |  |  |  |  |
| 1 | G-C-G-**A-**C-T-T-G-T-**G-**A-C-**G-C-**T-**T-**A-G |  | 0.449 | 0.462 | 1.00 |  |  |
| 2 | A-C-A-**A-**T-G-T-A-T-**G-**A-T-**G-C-**G-***G-***T-G |  | 0.174 | 0.173 | 1.07 (0.83 - 1.37) | 0.6 | 1 |
| 3 | G-C-G-**A-**C-T-C-G-T-**G-**G-C-**G-C-**T-**T-**A-G |  | 0.0724 | 0.0665 | 1.20 (0.85 - 1.70) | 0.31 | 1 |
| 4 | G-T-G-**A-**T-G-T-A-C-***A-***A-T-**G-C-**G-**T-**T-T |  | 0.0507 | 0.0622 | 0.86 (0.59 - 1.25) | 0.42 | 1 |
| 5 | G-C-G-***G-***C-G-T-A-C-**G-**A-T-***A-T-***G-***G-***T-T |  | 0.0470 | 0.0476 | 1.04 (0.69 - 1.57) | 0.84 | 1 |
| 6 | G-C-G-**A-**C-T-T-G-T-**G-**G-C-**G-C-**T-**T-**A-G |  | 0.0566 | 0.0512 | 1.17 (0.80 - 1.70) | 0.43 | 1 |
| 7 | G-C-G-**A-**C-G-T-A-C-**G-**A-T-**G-C-**G-**T-**T-T |  | 0.0157 | 0.0158 | 1.05 (0.52 - 2.15) | 0.88 | 1 |
| 8 | G-C-G-**A-**C-T-T-G-T-**G-**A-C-**G-C-**T-**T-**T-T |  | 0.0151 | 0.0119 | 1.27 (0.60 - 2.69) | 0.54 | 1 |
| Rare haplotypes | |  | 0.115 | 0.108 | 1.16 (0.83 - 1.63) | 0.39 | 1 |
| Global |  |  |  |  |  | 0.9 |  |
| Significant results at *P*<0.05 are in bold. For the five SNPs (rs10499051, rs7762395, rs4946933, rs4945815 and rs3800230) significantly associated with male longevity, alleles are in bold and alleles different from the corresponding alleles in Haplotype 1 (G-C-G-**A-**C-T-T-G-T-**G-**A-C-**G-C-**T-**T-**A-G) are in bold and italic. *P*c, *P* values after Bonferroni correction using the number of haplotypes tested (n = 8, 6, 8 for all subjects, men and women, respectively). | | | | | | | |

| **Table J. Haplotype analysis of male-longevity-associated SNPs in *FOXO3* in the Chinese Han population.** | | | | | | | |
| --- | --- | --- | --- | --- | --- | --- | --- |
| Haplotype | rs10499051-rs7762395- rs4945815-rs3800230 |  | Frequency | | OR (95% CI) | *P* | *P*c |
|  |  |  | LLIs | Controls |  |  |  |
| All subjects | |  |  |  |  |  |  |
| 1 | A-G-C-T |  | 0.671 | 0.662 | 1.00 |  |  |
| 2 | A-G-C-***G*** |  | 0.200 | 0.201 | 0.99 (0.81 - 1.21) | 0.93 | 1 |
| 3 | A-***A***-C-T |  | 0.0556 | 0.0691 | 0.81 (0.59 - 1.11) | 0.18 | 0.9 |
| 4 | ***G***-G-***T***-***G*** |  | 0.0614 | 0.0508 | 1.19 (0.85 - 1.66) | 0.31 | 1 |
| 5 | ***G***-G-C-T |  | 0.00850 | 0.0120 | 0.75 (0.33 - 1.72) | 0.49 | 1 |
| Rare haplotypes | |  | 0.00360 | 0.00550 | 0.52 (0.09 - 2.99) | 0.46 | 1 |
| Global |  |  |  |  |  | 0.46 |  |
| Men | |  |  |  |  |  |  |
| 1 | A-G-C-T |  | 0.641 | 0.671 | 1.00 |  |  |
| 2 | A-G-C-***G*** |  | 0.208 | 0.200 | 1.13 (0.72 - 1.79) | 0.6 | 1 |
| 3 | A-***A***-C-T |  | 0.0208 | 0.0548 | 0.44 (0.15 - 1.34) | 0.15 | 0.6 |
| 4 | ***G***-G-***T***-***G*** |  | 0.125 | 0.0578 | **2.30 (1.15 - 4.59)** | **0.019** | 0.076 |
| Rare haplotypes | |  | 0.00520 | 0.0170 | 0.34 (0.04 - 3.04) | 0.34 | 1 |
| Global |  |  |  |  |  | **0.028** |  |
| Women | |  |  |  |  |  |  |
| 1 | A-G-C-T |  | 0.678 | 0.660 | 1.00 |  |  |
| 2 | A-G-C-***G*** |  | 0.198 | 0.200 | 0.97 (0.78 - 1.20) | 0.76 | 1 |
| 3 | A-***A***-C-T |  | 0.0625 | 0.0725 | 0.85 (0.61 - 1.18) | 0.33 | 1 |
| 4 | ***G***-G-***T***-***G*** |  | 0.0488 | 0.0492 | 0.97 (0.66 - 1.43) | 0.88 | 1 |
| 5 | ***G***-G-C-T |  | 0.00990 | 0.0123 | 0.82 (0.35 - 1.92) | 0.64 | 1 |
| Rare haplotypes | |  | 0.00360 | 0.00510 | 0.59 (0.10 - 3.46) | 0.56 | 1 |
| Global |  |  |  |  |  | 0.89 |  |
| Significant results at *P*<0.05 are in bold. Alleles in bold and italic are different from the corresponding alleles in Haplotype 1 (A-G-C-T). *P*c, *P* values after Bonferroni correction using the number of haplotypes tested (n = 5, 4 and 5 for all subjects, men and women, respectively). | | | | | | | |

| **Table K. Haplotype frequencies of male-longevity-associated SNPs in *FOXO3* in the Chinese Han long-lived individuals and controls.** | | | | | | | | | | | |
| --- | --- | --- | --- | --- | --- | --- | --- | --- | --- | --- | --- |
| Haplotype | rs10499051-rs7762395-rs4945815-rs3800230 |  | LLIs | | |  | Controls | | |  | *P* (male LLIs vs female controls) |
|  |  |  | Frequency | | *P* |  | Frequency | | *P* |  |  |
|  |  |  | Men | Women |  |  | Men | Women |  |  |  |
| 1 | A-G-C-T |  | 0.641 | 0.678 |  |  | 0.671 | 0.660 |  |  |  |
| 2 | A-G-C-***G*** |  | 0.208 | 0.198 | 0.39 |  | 0.200 | 0.200 | 0.95 |  | 0.73 |
| 3 | A-***A***-C-T |  | 0.0208 | 0.0625 | 0.072 |  | 0.0548 | 0.0725 | 0.33 |  | **0.027** |
| 4 | ***G***-G-***T***-***G*** |  | 0.125 | 0.0488 | **0.0003** |  | 0.0578 | 0.0492 | 0.65 |  | **0.0005** |
| 5 | ***G***-G-C-T |  |  |  |  |  | 0.00940 | 0.0123 | 0.76 |  | 1 |
| Rare haplotypes | |  | 0.00520 | 0.0135 | 0.39 |  | 0.00760 | 0.00510 | 0.81 |  | 0.83 |
| Global |  |  |  |  | **0.0005** |  |  |  | 0.92 |  | **0.00028** |
| Significant results at *P*<0.05 are in bold. Alleles in bold and italic are different from the corresponding alleles in Haplotype 1 (A-G-C-T). | | | | | | | | | | | |
